# ALX148 blocks CD47 and enhances innate and adaptive antitumor immunity with a favorable safety profile

**DOI:** 10.1371/journal.pone.0201832

**Published:** 2018-08-22

**Authors:** Steven E. Kauder, Tracy C. Kuo, Ons Harrabi, Amy Chen, Emma Sangalang, Laura Doyle, Sony S. Rocha, Sangeetha Bollini, Bora Han, Janet Sim, Jaume Pons, Hong I. Wan

**Affiliations:** ALX Oncology, Burlingame, CA, United States of America; Center for Cancer Research, UNITED STATES

## Abstract

CD47 is a widely expressed cell surface protein that functions as an immune checkpoint in cancer. When expressed by tumor cells, CD47 can bind SIRPα on myeloid cells, leading to suppression of tumor cell phagocytosis and other innate immune functions. CD47-SIRPα signaling has also been implicated in the suppression of adaptive antitumor responses, but the relevant cellular functions have yet to be elucidated. Therapeutic blockade of the CD47 pathway may stimulate antitumor immunity and improve cancer therapy. To this end, a novel CD47-blocking molecule, ALX148, was generated by fusing a modified SIRPα D1 domain to an inactive human IgG1 Fc. ALX148 binds CD47 from multiple species with high affinity, inhibits wild type SIRPα binding, and enhances phagocytosis of tumor cells by macrophages. ALX148 has no effect on normal human blood cells *in vitro* or on blood cell parameters in rodent and non-human primate studies. Across several murine tumor xenograft models, ALX148 enhanced the antitumor activity of different targeted antitumor antibodies. Additionally, ALX148 enhanced the antitumor activity of multiple immunotherapeutic antibodies in syngeneic tumor models. These studies revealed that CD47 blockade with ALX148 induces multiple responses that bridge innate and adaptive immunity. ALX148 stimulates antitumor properties of innate immune cells by promoting dendritic cell activation, macrophage phagocytosis, and a shift of tumor-associated macrophages toward an inflammatory phenotype. ALX148 also stimulated the antitumor properties of adaptive immune cells, causing increased T cell effector function, pro-inflammatory cytokine production, and a reduction in the number of suppressive cells within the tumor microenvironment. Taken together, these results show that ALX148 binds and blocks CD47 with high affinity, induces a broad antitumor immune response, and has a favorable safety profile.

## Introduction

A central question in the study of cancer is why the immune system sometimes fails to mount an effective antitumor response despite possessing the components needed to do so. One cause of this failure has become clear with the identification of checkpoint pathways, which are co-opted by tumors to inhibit their elimination by immune cells. This phenomenon has been best described for the adaptive component of the immune response, where cytotoxic T cell activity is suppressed by checkpoint signals originating from tumor and other cells in the tumor microenvironment [1]. In the clinic, the CTLA-4 and PD-1 T cell checkpoint pathways have been validated as therapeutic targets, with their blockade leading to enhancement of the patient’s immune response and, in some cases, durable antitumor efficacy across several tumor types [2–4].

The CD47 pathway is an additional checkpoint that can suppress antitumor immunity [5, 6]. In contrast to previously identified checkpoint pathways that target the adaptive arm of the immune response, this pathway suppresses the activity of innate immune cells [7, 8]. CD47 is expressed on the surface of a broad range of cell types [9, 10], and this expression protects healthy cells from macrophage-mediated phagocytosis by interacting with its receptor, signal regulatory protein-α (SIRPα) [11, 12]. Engagement of SIRPα triggers signaling through SIRPα immunotyrosine inhibitory motifs (ITIMs), which inhibits phagocytosis and other components of macrophage function [13–21].

Analyses of human tumor tissue have implicated CD47 in cancer. High levels of CD47 expression have been observed in a variety of hematological and solid tumors [5, 22], and elevated CD47 expression is an adverse prognostic indicator for survival [22–25]. These findings indicate that tumor cells may utilize the CD47 pathway to evade macrophage surveillance. One component of this surveillance is Antibody-Dependent Cellular Phagocytosis (ADCP), in which antitumor antibodies initiate phagocytosis by binding tumor cells and engaging macrophage Fc gamma (Fcγ) receptors [26–28]. Blockade of the CD47-SIRPα interaction enhances ADCP of tumor cells [24, 29–32], demonstrating that if unchecked, CD47 expression can protect tumor cells from macrophage phagocytosis. Similarly, CD47 blockade in mouse studies inhibits the growth of human tumor xenografts and promotes survival [22, 24, 25, 30, 33]. Notably, these xenograft studies utilized immunocompromised mice that lack most immune cell types other than macrophages. Thus, while these studies demonstrated that CD47 blockade activates a macrophage-mediated antitumor response, they were incapable of identifying the roles played by other cells in the context of an intact immune system.

To better understand the full range of responses induced by CD47 blockade, CD47 function has been disrupted in immunocompetent mice [34–36]. These studies have shown dendritic cells (DCs) and T lymphocytes to be important components of the resultant antitumor response. DCs express SIRPα, and inhibition of the CD47-SIRPα interaction in a model using exogenous sheep red blood cells triggered DC activation, leading to enhanced T cell responses [37]. Furthermore, studies of syngeneic tumors in immunocompetent mice have demonstrated that disruption of CD47 signaling can induce macrophage, DC, and T cell-mediated antitumor responses. In fact, both DCs and T cells have been shown to be essential for the CD47-mediated antitumor response [34, 38]. Further evidence for interplay between innate and adaptive immunity in response to CD47 blockade comes from a combination study of CD47 and PD-L1 blocking agents. The activation of T cells by PD-L1 inhibition was required to maximize the effect of CD47-SIRPα disruption [39]. Thus, the therapeutic targeting of the CD47 pathway can induce a broad antitumor response involving both innate and adaptive immunity. While depletion studies have shown the importance of cells such as DCs and T lymphocytes for this response, the cellular functions activated by these agents are unknown.

Because tumor cells potentially utilize the CD47 pathway to evade both innate and adaptive components of immune surveillance, therapeutic blockade of this pathway may activate antitumor immunity and improve cancer therapy. For this reason, a variety of antibodies and fusion proteins targeting the CD47 pathway are in clinical development [30, 31, 40]. Notably, the majority of these molecules share a common design in that they are essentially bifunctional. First, they bind CD47, blocking its interaction with SIRPα and suppressing downstream signals that inhibit phagocytosis. Second, they contain Fc domains that engage macrophage Fcγ receptors, inducing phagocytosis. These molecules induce phagocytosis *in vitro* and are efficacious against human tumor cell xenografts in mice. However, normal healthy blood cells also express high levels of CD47, making them potential targets for these molecules. In fact, dose-limiting toxicities related to anemia and thrombocytopenia have been reported for several of these molecules in both animal and clinical oncology studies [31, 41, 42]. These toxicities underscore the need to ensure that CD47 blockade activates immune responses against tumor cells while sparing normal healthy cells.

For the reasons discussed above, a safer approach to targeting CD47 is needed to achieve the therapeutic potential of CD47 blockade. ALX148 was generated as an investigational anticancer therapeutic to safely target CD47 in humans, enhancing antitumor immunity while having minimal effects on normal cells. ALX148 is comprised of the N-terminal D1 domain of SIRPα, which binds CD47 [43, 44], fused to a modified human IgG1 Fc domain. The SIRPα D1 portion contains amino acid substitutions previously shown to dramatically increase affinity for CD47 [32], thus enabling the blockade of wild-type SIRPα binding. Furthermore, this modified SIRPα domain is designed to bind cynomolgus monkey and murine CD47 with high affinity, allowing direct investigation of ALX148 toxicology and function in these species.

The Fc portion of ALX148 confers increased molecular mass and interaction with the neonatal Fc receptor, both of which are associated with extended pharmacokinetics. In ALX148, this domain has a unique set of amino acid substitutions designed to eliminate binding to human Fcγ receptors and complement C1q protein [45–48]. The absence of effector function should prevent ALX148 from engaging Fcγ receptors and targeting normal cells for phagocytosis, differentiating ALX148 from other CD47-blocking molecules that have active Fc domains.

Here, we describe results of preclinical studies of ALX148. This investigational drug potently blocked the CD47-SIRPα interaction and enhanced antibody dependent phagocytosis. In murine models utilizing human tumor xenografts, ALX148 improved the efficacy of targeted antitumor antibodies that are currently in clinical use. Because ALX148 binds murine CD47 with high affinity, effects on antitumor efficacy and immune responses were evaluated in syngeneic murine tumor models. In these models, ALX148 enhanced the antitumor activity of multiple immunotherapeutic agents. Furthermore, as a single agent and in combination with these agents, ALX148 enhanced DC, macrophage, and T cell responses associated with antitumor immunity. These findings demonstrate that ALX148, via CD47 blockade, elicits immune responses that bridge innate and adaptive immunity and are associated with enhanced antitumor efficacy. Consistent with the absence of Fc effector function, in rodent and non-human primate studies no ALX148-related adverse events were observed in hematological or other parameters. These results indicate that ALX148 has the potential to be an effective anti-cancer therapeutic in humans while maintaining a favorable safety profile. Therefore, ALX148 is currently in clinical development for multiple oncology indications.

## Materials and methods

### Molecular cloning, protein expression and purification

Wild type SIRPα, CV1 [32], human IgG1 Fc, human IgG1 Fc variants, and human IgG4 Fc with S228P and L235E substitutions (hIgG4 PE), rituximab, hexahistidine-tagged CD47, trastuzumab, daratumumab, and anti-PD-L1 (based on atezolizumab, with murine IgG1 Fc) were synthesized (ATUM) based on publicly available sequences in CMV promoter-driven expression plasmids for mammalian cell expression. The SIRPα domains of ALX148 incorporate amino acid substitutions that enhance affinity for CD47 [32]. This was fused to human IgG1 Fc with modifications that eliminate effector function [45–48]. ALX377 was generated by fusing the SIRPα domains of ALX148 to unmodified human IgG1 Fc. ALX180 was generated using site directed mutagenesis to introduce V33R and Q52R substitutions in wild type SIRPα fused to a modified IgG1 Fc. ALX222 was generated by fusing CV1 to human IgG1 Fc (GeneArt Seamless PLUS Cloning and Assembly Kit, Invitrogen). ALX217 was made by fusing the SIRPα domain from ALX148 to human IgG4 S228P, S235E. Constructs were expressed in Expi293 cells (Invitrogen) at 3 ml to 1L scale in deep well blocks or shake flasks. Expression cultures were grown five days at 37°C in 8% CO_2_ while shaking. Supernatants were harvested via centrifugation and sterile filtered. Proteins were affinity purified utilizing either an Akta Avant (GE Healthcare) or Amicon Pro Purification Device (Millipore Sigma). Antibodies and Fc fusion proteins were bound to MabSelectSure LX resin (GE Healthcare), washed with 1x phosphate-buffered saline (1xPBS), eluted with 0.1 M citric acid pH 3.3, neutralized with 10% v/v 1 M sodium phosphate pH 8, then dialyzed into PBS. CD-47 His was bound to a chelating sepharose resin, step eluted with 5, 40, and 300mM Imidazole with a running buffer of 25mM phosphate pH 7.5, 500mM NaCl followed by a gel filtration polishing step.

The nucleotide coding sequence for ALX148 was subcloned into an expression vector under the control of the hEF-1-alpha promoter coupled to a CMV enhancer. A stable CHO cell line expressing ALX148 was generated and used for production of ALX148. Purification of ALX148 was carried out using protein A affinity chromatography similar to conventional antibody purification. The final purified material was concentrated into storage buffer and stored at -80°C until use.

### Cell lines

OE19, DLD-1, Raji, Z138, Daudi, Jurkat, MM1.R, and CT26 cells were obtained from the American Type Culture Collection. DLD-1 GFP Luciferase cells [32] were obtained from K. Christopher Garcia, Stanford University. CT26-M/H HER2 cells were generated by infecting CT26 cells with a lentivirus vector encoding a chimera of mouse and human HER2 transmembrane and extracellular domains. MC38 cells were obtained from MuriGenics. Cell lines were cultured according to standard protocols.

### Animals

All mouse experiments were conducted according to protocols approved by Institutional Animal Care and Use Committee of Alexo Therapeutics (protocol number AL-002-2017 for hematology studies and protocol number AL-001-2017 for tumor studies). BALB/C, C57BL/6, NOD-SCID and CD-1 animals were purchased from Charles River Laboratories International (Hollister, CA). All animals were housed in a pathogen free facility located in the vivarium at Alexo Therapeutics, South San Francisco in accordance to IACUC guidelines. Animals used for all studies were 6–8 weeks old.

The non-human primate CD47 occupancy study was conducted at Charles River Laboratories in accordance with standard operating procedures and with the approval of the IACUC. Animals were housed in stainless-steel cages. Primary enclosures were as specified in the USDA Animal Welfare Act (9 CFR, Parts 1, 2 and 3) and as described in the Guide for the Care and Use of Laboratory Animals. The targeted conditions for animal room environment were temperature of 64F to 84F, humidity of 30% to 70%, ventilation of greater than 10 air changes per hour with 100 percent fresh air, light cycle of 12 hour light/12 hour dark. Purina Certified Primate Diet No. 5048 was provided daily in amounts appropriate for the size and age of the animals. Municipality tap water was processed through a reverse osmosis filter and passed through UV light treatment. Animals received fruit and/or vegetable supplements a minimum of four times per week. Food distribution by means of human interaction was provided a minimum of two times per week. At least one interior (in cage) enrichment toy was provided for each animal at all times. Animals were socialized continuously unless prohibited due to incompatibility, medical concerns, or study activities, up to three animals of the same sex and same dosing group together.

The non-human primate toxicity study was conducted at Covance Laboratories. All procedures were approved by the Institutional Animal Care and Use Committee (IACUC) of Covance Laboratories, Madison WI (Approval number 8333311). Procedures were also in compliance with the Animal Welfare Act (P.L. 89–544), the Guide for the Care and Use of Laboratory Animals (National Research Council of the National Academies), and the Office of Laboratory Animal Welfare. Animals were housed in stainless steel cages. Where possible, animals were socially housed by sex: up to three animals per cage. Certified Primate Diet #5048 (PMI Nutrition International) was provided one or two times daily. Water was provided ad libitum. Environmental controls for the animal room were set to maintain 20 to 26°C, a relative humidity of 30 to 70%, a minimum of eight air changes/hour, and a 12-hour light/12-hour dark cycle. Animals were given various cage-enrichment devices; fruit, vegetable, or dietary enrichment (that do not require analyses). Animals were commingled in accordance with Covance standard operating procedures.

### Affinity determination

All experiments were performed at 25°C and 37°C using a SPR-based ProteOn XPR36 biosensor (BioRad) equipped with GLC sensor chips. Running buffer was PBS pH 7.4 with 0.01% Tween-20 (PBST+). All analytes (human, cynomolgus monkey, mouse and rat CD47-ECD) were used at their nominal concentrations as determined by A280 absorbance and molar extinction coefficient. Analytes were injected in a “one-shot” kinetic mode [49].

The interactions of ALX148 with human, cynomolgus monkey, mouse and rat CD47-ECD were analyzed by flowing CD47-ECD over ALX148 immobilized (200–400 RUs) on GLC chips using Proteon Amine Coupling Kit (Biorad). GLC chips were activated with EDAC/Sulpho-NHS 1:1 (Biorad) diluted 1:500 for 300 sec at 25 μL/min. ALX148 proteins were diluted to 80 nM concentration in 50 mM sodium acetate buffer pH 4.5 and immobilized to the chip at 30 μl/min for 50 seconds. Chips were inactivated with ethanolamine for 300 sec at 25 μL/min.

Analytes (CD47-ECD) were injected in a “one-shot” kinetic mode at nominal concentrations of 300 nM or 100 nM with 3-fold serial dilutions. Association times were monitored for 60 seconds, and dissociation times were monitored 1200 seconds for human and cynomolgus monkey, 600 seconds for mouse and rat CD47. The surfaces were regenerated with a 2:1 v/v blend of Pierce IgG elution buffer and 4M NaCl.

Biosensor data were double-referenced by subtracting the interspot data (containing no immobilized protein) from the reaction spot data (immobilized protein) and then subtracting the response of a buffer “blank” analyte injection from that of an analyte injection. Double-referenced data were fit globally to a simple Langmuir model and the KD value was calculated from the ratio of the apparent kinetic rate constants (KD = kd/ka).

### ALX148 binding on cells and blockade of SIRPα binding to CD47 cells

For detection of cell binding, ALX148, ALX180 and SIRPα-IgG1 Fc fusion protein (ALX234) were fluorescently labeled with the Alexa Fluor 647 Protein Labeling Kit (Thermo Fisher Scientific) according to the manufacturer’s instructions. 100,000 cells per well in staining buffer (PBS, 0.5% BSA or 2%FBS) were plated in 96 well plates (Falcon).

To detect ALX148 binding to cells, Alexa Fluor 647-labeled ALX148 or negative control ALX180 at a final concentrations of 20 nM were added to cells in 50 μl volume of FACS buffer (PBS + 0.5% BSA) supplemented with a cocktail of human Fc block (Miltenyi Biotec) and human AB serum (Corning). After a 15 minute incubation on ice, cells were washed twice in staining buffer, stained with a fixable Live/Dead stain (Invitrogen), followed by a final wash and fixed in 0.5% formaldehyde.

To block SIRPα binding to CD47 on Jurkat cells, ALX148 or ALX180 at a concentration of 12.5 nM were added to cells. After a 15 minute incubation on ice, cells were washed in FACS buffer and incubated with labeled ALX234 at a concentration of 2.5 μM. Wild type SIRPα binding is indicated by the geometric mean fluorescence for Alexa Fluor 647.

Cells were analyzed on a FACS Canto II (BD Biosciences), with subsequent data analysis and histogram plotting using Flowjo 10.7.

### Human FcRn and FcγRs binding

All experiments were performed at 25°C using a SPR-based ProteOn XPR36 biosensor (BioRad, Inc, Hercules, CA) equipped with GLC sensor chips. The running buffer was PBS pH 7.4 with 0.01% Tween-20 (PBST+) and PBS pH 5.8 with 0.01% Tween-20 for FcRn interactions. All analytes were used at their nominal concentrations as determined by dividing the manufacturer-provided protein amount by volume of dissolution buffer. Analytes were injected in a “one-shot” kinetic mode.

The interactions of ALX proteins with hFcγRs and hFcRn were analyzed by flowing the receptors over ALX proteins captured on a human CD47 GLC chip. Approximately 250 RU of human CD47 ECD were immobilized on a GLC chip using amine chemistry (following manufacturer instructions). ALX proteins were captured for 300 seconds at 25 μl/min at 40 nM in PBST+ (300–400 RUs). Analytes (hFcγRs and hFcRn) were injected in a “one-shot” kinetic mode at nominal concentrations of 0, 61, 185, 555, 1666, and 5000 nM. Association times were monitored for 200s and dissociation times were monitored for 120s (except for hFcγRI where the dissociation time was 600s). The surfaces were regenerated with a 2:1 v/v blend of Pierce elution buffer/4M NaCl.

Biosensor data were double-referenced by subtracting the interspot data (containing no immobilized protein) from the reaction spot data (immobilized protein) and then subtracting the sensograms of blank reference channel. Double-referenced data were fit to an equilibrium analysis using a simple binding isotherm. For Fcs with strong binding to hFcγRI, data were also fit globally to a simple Langmuir model and the KD value was calculated from the ratio of the apparent kinetic rate constants (KD = kd/ka).

### Human complement C1q binding

Immulon 4HBX ELISA plates (Thermo Fisher Scientific) were coated overnight with bovine serum albumin (BSA, Thermo Fisher Scientific), ALX217, ALX148, or ALX222 at 5 μg/ml in PBS. Plates were blocked in assay buffer pH 6.0 (PBS with 0.5% BSA, 0.05% Tween-20, 0.25% CHAPS, 5mM EDTA, 0.35M NaCl, Teknova) and washed in 1x TBST (Teknova). Plates were incubated with complement C1q (Quidel) for 1 hour at room temperature and washed with wash buffer. Plates were then incubated for 1 hour at room temperature with HRP-conjugated sheep anti-complement C1q antibody (Thermo Fisher Scientific) at 2 μg/mL and washed, followed by the addition of 3,3’,5,5’-tetra-methylbenzidine peroxidase substrate (Thermo Fisher Scientific) and incubation for 10 minutes. The reaction was terminated with 0.16M sulfuric acid (Thermo Fisher Scientific) and the resulting absorbance was measured at 450 nm with a reference of 570 nm using a SpectraMax i3 plate reader (Molecular Devices).

### ADCC reporter bioassay

The assay was performed with the ADCC Reporter Bioassay Core Kit (Promega) using Raji cells as targets according to the manufacturer’s instructions. Black walled, clear bottomed 96 well assay plates (Corning) were incubated with test proteins, 12,500 Raji cells, and 75,000 Jurkat Effector Cells for 6 hours at 37°C. Bio-Glo luciferase assay reagent was added and luminescence was read in a SpectraMax i3 plate reader (Molecular Devices). IgG1, k antibody (Southern Biotech) was used as a negative control.

### Hemagglutination assay

Human erythrocytes were isolated from whole blood buffy coats (Stanford University Blood Center) by purification with Ficoll-Paque Plus (GE Healthcare) and subsequent washing of erythrocyte pellets in PBS. 4 million erythrocytes in PBS were plated per well in polypropylene 96 well plates (Corning). Plates were incubated with ALX148, B6H12 (Thermo Fisher Scientific), CC2C6, or PBS for four hours at room temperature. Plate images were captured as unmodified jpeg images.

### Phagocytosis assay

CD14^+^ cells were purified from Trima residuals (Blood Centers of the Pacific) with Ficoll-Paque Plus and negative selection (Monocyte Isolation Kit II, Miltenyi Biotec) according to the manufacturers’ protocols. Monocyte-derived macrophages were made by seeding 10 million CD14^+^ cells into 150 mm tissue culture dishes (Corning) in growth medium supplemented with 10% human AB serum (Corning). Cells were cultured for seven to eleven days. Adherent cells were detached from culture plates with TrypLE Select (Thermo Fisher Scientific). Where necessary, target cells were labeled with the Celltrace CFSE Cell Proliferation kit (Thermo Fisher Scientific) according to the manufacturer’s instructions.

100,000 target cells and 50,000 monocyte-derived macrophages were incubated in ultra-low attachment U-bottom 96 well plates (Corning) with ALX148 or ALX180 and the corresponding tumor-specific antibody for two hours at 37° C. For DLD-1 GFP Luciferase cells, cetuximab (Absolute Antibody) was added at a concentration of 1 μg/ml. For OE19 cells, trastuzumab (Absolute Antibody) was added at a concentration of 0.01 μg/ml. For MM.1R, daratumumab (Alexo Therapeutics) was added at a concentration of 1 μg/ml. For Daudi cells, obinutuzumab (Roche) was added at a concentration of 0.1 μg/mL. IgG1, k isotype control antibody was purchased from SouthernBiotech.

For flow cytometry, cells were incubated in human FcR Blocking Reagent (Miltenyi Biotec) and stained with fluorochrome-labeled antibodies against CD33 (clone WM53, Biolegend) and CD206 (clone 15–2, Biolegend). To eliminate macrophage/target cell adhesion from analyses, antibody against CD326 (clone 9C4, Biolegend) was included for DLD-1 GFP Luciferase and OE19 cells, antibody against CD138 (clone MI15, Biolegend) was included for MM1.R cells, and antibodies against CD20 (clone 2H7, Biolegend) was included for Daudi cells. Furthermore, a pulse geometry gate of forward scatter signal area vs height was used to select for single cells. Fixable viability dye (Thermo Fisher Scientific) was used to identify live cells. Cells were analyzed on a FACS Canto II flow cytometer (BD Biosciences) with subsequent analysis using FlowJo software. Percent phagocytosis indicates the percentage of viable CD33^+^CD206^+^ macrophages that stain negative for target cell markers and positive for GFP or CFSE. Where applicable, 4 parameter fit curves were generated with Prism 7 software (GraphPad).

For fluorescence microscopy, cells were washed twice with PBS after a two hour incubation of macrophages and DLD-1 cells in the presence of various treatments. Washed cells were resuspended in PBS and 60,000 cells in 25ul were spread on a slide (ONCYTE) to air dry at room temperature. Cells were fixed with 4% paraformaldehyde for 15 minutes at room temperature, followed by two washes with PBS. Slides were partially dried and 50ul of antifade mount (ProLong) and coverslip were used to seal the cells. CFSE labeled target cells and macrophages were visualized by bright field and fluorescence using an Axioimager upright microscope (Zeiss) with a 470 nM eGFP filter at 20x magnification. Digital images were taken with a AxioCam Hrm (Zeiss) and digitally merged using Fiji software [50].

### Murine hematology assay

Animals were monitored for adverse clinical signs and humane endpoints were applied according to the Institutional Animal Care and Use Committee of Alexo Therapeutics protocol number AL-002-2017. Animals were observed for a minimum of an hour following dosing, then minimally once daily, increasing if any clinical abnormalities were observed. Any observed adverse clinical signs were described and documented in the study record and included time of onset relative to dose administration and if appropriate, time to recovery. Animals with severe clinical signs, with no improvement, or the animals not anticipated to recover before the next scheduled time point or dose administration were euthanized. Body weights were obtained pre-dose and then, minimally, once a week (increasing to daily if 15% body weight loss is evidenced) for the duration of the study. Any animal that appeared moribund (lethargic, hunched posture, cold to touch, etc.) and/or had a loss of ≥20% in body weight as compared to age-matched, nonmanipulated cohorts was euthanized.

ALX148 and ALX377 were administered intravenously to female CD-1 mice via tail vein on day 0. Mice were assigned to two groups of ten mice and were treated with 30 mg/kg ALX377 or 30 mg/kg ALX148. For complete blood counts (CBC) analysis, blood was collected via tail vein into K2EDTA microcapillary tubes (Heska) and hematologic parameters were evaluated using a HeskaView analyzer. Every time point contains four-five mice/treatment group at the following time points: Day -5, 72 (Day 3), 120 (Day 5), and 240 (Day 12) hours post dose.

### In vivo tumor studies

Isoflurane anesthesia were used on mice to eliminate or minimize pain and distress during tumor impantation. CT26 and MC38 colon tumor cells (0.5×10^6^) were resuspended in 100 μl PBS or RPMI and implanted subcutaneously into the flank of female BALB/c and C57BL/6 mice, respectively. For the MC38 immunophenotyping study, 2 x 10^6^ cells were implanted. OE19 (5×10^6^), Raji (5×10^6^), and Z138 (5×10^6^) tumor cells were resuspended in 100 μl 1:1 PBS:matrigel (Corning) and implanted subcutaneously in the flank of NOD-SCID mice. When tumors reached an average volume of 50-250mm^3^ (depending on the model), as calculated with the formula volume = (width^2^ x length)/2, mice were randomized into treatment groups. All treatments were dosed intraperitoneally (i.p.). For xenograft models in NOD-SCID mice, rituximab, obinutuzumab, and trastuzumab were dosed twice/week for three weeks. Treatment groups for syngeneic models vary and are as described in figure legends. Mice were treated with 5 mg/kg anti-PD-1 (BioXcell, clone RMP1-14) three times, five days apart, 2 mg/kg or 3 mg/kg anti-PD-L1 (murine IgG1, Alexo Therapeutics) three times, five days apart and 0.1 mg/kg anti-4-1BB (BioXcell, clone LOB12.3) two times, five days apart. Mice in the MC38 immunophenotyping study were dosed once a week with anti-PD-L1.

Mice were observed daily with a minimum of weekly tumor measurements when palpable tumors were present and daily once tumor volume reaches a maximum of 1500 mm^3^ or if animals are showing abnormal signs or body weight changes by 15%. Animals were monitored for adverse clinical signs and humane endpoints were applied according to the Institutional Animal Care and Use Committee of Alexo Therapeutics protocol number AL-001-2017. Animals were euthanized by CO_2_, followed by cervical dislocation at the end of the study or immediately during the study when weight loss ≥20% of baseline, tumor greater than 2000 mm^3^, tumor interferes with normal bodily functions, discharge from tumor, respiratory or central nervous system abnormalities, self-mutilation, hypothermia or veterinarian determines that the animal should be euthanized for humane concerns. No unexpected deaths were observed. The length of the study depended on the model. For survival endpoints, the duration of MC38 and CT26 syngeneic tumor model studies were between 35–45 days and for Raji xenograft model was around 50–55 days. All animals in the studies were euthanized due to tumor size greater than 2000 mm^3^ or at the end of the study. The number of mice used per study is the same as the number of mice euthanized. All studies included 40 mice except for CT26 (ALX148 + anti-PD-1 combo study), which include 36 mice.

### CD47 occupancy assays

BALB/c mice were implanted subcutaneously with 0.5 million CT26-M/H HER2 tumor cells in the right flank. Thirteen days after implantation, mice were randomized into groups with equivalent tumor sizes and administered PBS or unlabeled ALX148 at doses of 10 or 30 mg/kg via intraperitoneal injection. At 1, 4, or 8 days after administration, 100 μl blood was removed by cheek bleed with a Goldenrod animal lancet (MEDIpoint) and centrifuged in serum separation tubes (Becton Dickinson) to isolate serum for ALX148 ELISA. Mice were then euthanized and tumors and spleen removed. Tumors were approximately 500 to 900 mm^3^ in size at the time of euthanasia. Single cell suspensions were made from tumors (Tumor Dissociation Kit, mouse, Miltenyi Biotec) using 1/10 the recommended volume of enzyme R and from spleen by mechanical dissociation, with red blood cell lysis using ACK buffer (Thermo Fisher Scientific). Five million cells were incubated in 5% mouse serum (Jackson Immunoresearch), then stained with fluorochrome-labeled antibodies against CD3 (clone 145-2C11, Biolegend), CD4 (clone RM4-5, Biolegend), and trastuzumab (Alexo Therapeutics) and Fixable Viability Dye to identify live cells. CD47 occupancy was determined by including labeled ALX148 in the stain cocktail. Cells were analyzed on a FACS Canto II flow cytometer with subsequent analysis using FlowJo 10.4 software.

Female cynomolgus monkeys (n = 2) were administered a single intravenous dose of ALX148 at 10 and 30 mg/kg along with a single intravenous dose of 0.05 mg/kg rituximab. From 75 μl blood, red blood cells were lysed using ACK buffer and remaining cells were treated with FcR blocking reagent and stained with fluorochrome-labeled antibodies against CD3 (clone SP34-2, BD Biosciences), CD4 (clone OKT4, Biolegend), and CD8 (clone SK1, Biolegend). CD47 occupancy was determined by including labeled antibody against CD47 (clone B6H12).

Cells were analyzed on a FACS Canto II flow cytometer with subsequent analysis using FlowJo 10.4 software. To calculate percent CD47 occupancy, the geometric mean fluorescence for labeled ALX148 or B6H12 binding was determined. Values were normalized to those from naïve animals, with the CD47 signal from naïve animals being zero percent occupancy and the absence of a CD47 signal being one hundred percent occupancy.

### ALX148 serum ELISA assays

Immulon 96 well ELISA plates (Thermo Fisher Scientific) were coated overnight with hexahistidine-tagged CD47 (Alexo Therapeutics) in PBS. Plates were washed with Tris-Buffered Saline Tween-20 (TBST, 25 mM Tris, 0.15 M Nacl, 0.05% Tween-20, pH 7.5) and blocked for 1 hour with assay buffer (PBS, 1% BSA, 0.05% Tween-20, 0.25% CHAPS, 5 mM EDTA, 0.35 M NaCl). Serum samples diluted a minimum of 1:50 in assay buffer or ALX148 standard curve protein (two-fold serial dilutions in assay buffer from 250 to 2 ng/ml for mouse or 500 to 3.9 ng/ml for monkey samples) were added to blocked plates for 1 hour. Plates were washed with TBST. Mouse samples were incubated for 1 hour with HRP-conjugated goat anti-human IgG (H+L) antibody (Bethyl) and washed with TBST. Monkey samples were incubated for 1 hour with biotinylated goat anti-human IgG (H+L) antibody (Bethyl), washed with TBST, incubated for 30 minutes with HRP-conjugated Avidin D (Vector), and washed with TBST. All plates were incubated with 1-Step Ultra TMB ELISA solution (Thermo Fisher Scientific) and the reaction was stopped with 0.16 M sulfuric acid solution (Thermo Fisher Scientific). Plates were read at an O.D. of 450nm with a background reference reading at 570nm on a SpectraMax i3 plate reader (Molecular Devices). Protein concentrations of serum samples were interpolated from the ALX148 standard curve with a 4 parameter fit curve using Prism software (GraphPad).

### Immune-cell phenotyping

Immune-cell phenotyping and cytokine assessment were performed 10 days post- treatment injection in CT26 model and three days post-last dose in MC38 model. Spleens were harvested and processed into single-cell suspension in ice-cold PBS, lysed with ACK lysis buffer (Gibco), washed twice and re-suspended in PBS supplemented with 2% FBS. Tumor-derived single-cell suspensions were prepared using either the Tumor Dissociation Kit, mouse and the GentleMACS Dissociator according to the manufacturer’s instructions except 1/10 of recommended reagent R was used or a cocktail of Collagenase I (Worthington), IV (Sigma) and DNAse (Sigma) for 45 min at 37°C.

Cell counts were performed using ViCell counter (Beckman Coulter) for spleen and trypan blue exclusion with hemacytometer for tumor. Aliquots of 1–3 x 10^6^ cells were either used for cell-surface antigen staining or stimulation for cytokine assessment. For surface staining, cells were stained with LIVE/DEAD fixable dye (Thermo Fisher), followed by mouse Fc-block (Biolegend) and subsequently stained with antibodies according to cell-type specific antibody panels for at least 30 min at 4°C.

Tumor and splenic suspensions were stimulated *ex vivo* with 10 μg/mL AH1 peptide (Anaspec), a CT26 tumor antigen, in RPMI supplemented with 10% FBS for 4 hours at 37°C, 5% CO_2_ in the presence of Golgi-Stop (BD). Identification of CT26 tumor antigen-specific CD8+ T cells was performed using PE- or APC-conjugated AH-1 MHC class 1 tetramer (MBL MuL gp70 SPSYVYHQF) on cells directly from the spleen or on AH-1 stimulated tumor and splenic suspensions. *Ex vivo* cytokine assessment was either performed following AH1 peptide or PMA/ionomycin stimulation.

For PMA/ionomycin *ex-vivo* stimulation, cells were plated at 1–1.5 x 10^6^ cells/well in complete RPMI comprised of 10% heat-inactivated FBS, 2% Pen/Strep, 1% Glutamax, 1% MEAA, 1% sodium pyruvate, 25 mM HEPES and 5μM ß-mercaptoethanol supplemented with 50 ng/mL PMA (Fisher Scientific) and 1μM ionomycin (Sigma) in the presence of Golgi-Stop for at least 4 hours at 37°C, 5% CO_2_.

For tumor infiltrating macrophage phenotyping, single-cell suspensions were rested overnight in DMEM + 10% FBS + 1% Pen/Strep and stained for flow cytometry. M1 and M2 macrophages were determined according to the following markers- M1: CD45^+^CD11b^+^CD38^+^EGR2^-^; M2: CD45^+^CD11b^+^CD38^-^EGR2^+^. Fluorescence-minus-one (FMO) controls were applied for all intracellular staining.

### Flow cytometry antibodies

Antibodies used to characterize cell surface and intracellular proteins were purchased from Biolegend, BD Biosciences or eBioscience. Live cells were separated from the dead with LIVE/DEAD Fixable Viability Dye (Thermo Fisher Scientific). The following antibodies were used: anti-CD86 (GL-1), anti-I-A/I-E (M5/114.15.2), anti-CD8a (53–6.7), anti-33D1 (33D1), anti-CD11c (N418), anti-CD45 (30-F11), anti-CCR7 (4B12), anti-CD4 (RM4-5), anti-CD3e (145-2C11), anti-CD25 (PC61), anti-FOXP3 (150D), anti-CTLA4 (UC10-4B9), anti-KLRG1 (2F1/KLRG1), anti-CD62L (MEL-14), anti-Granzyme B (QA16A02), anti-KI67 (16A8), anti-CD44 (IM7), anti-B220 (RA3-6B2), anti-CD11b (M1/70), anti-Ly6C (HK1.4), anti-CD38 (90), anti-iNOS (CXNFT), anti-EGR2 (erongr2), anti-TNF (MP6-XT22), and anti-IFNγ (XMG1.2). Intracellular staining was performed using FoxP3 Fixation/Permeabilization Kit per manufacturer’s instructions (Thermo Fisher Scientific).

### Non-human primate toxicity study

Study was conducted at Covance Laboratories (Madison, WI, USA) in accordance with the standard operating procedures and Good Laboratory Practices (GLP). Experimentally, naïve, social housed male and female cynomolgus monkeys of Chinese origin (n = 3/sex/group, 2–4 years of age and weighing 2–5 kg at the onset of the study) were administered ALX148 on days 1, 8, 15, 22, and 29 by a slow bolus IV injection at doses of 0, 10, 30, and 100 mg/kg. For the 0, 10, and 100 mg/kg/week dose levels, reversal of potential toxicity was evaluated following a 4-week recovery phase (2/sex/group). Standard toxicological parameters were evaluated including toxicokinetics, immunogenicity, cytokine analysis, and hematology parameters. Complete histopathology was evaluated upon completion of the dosing and recovery period.

PK parameters were determined from individual animal data using noncompartmental analysis (WinNonlin).

### Statistical analysis

Results are expressed as mean +/- SEM unless otherwise noted. Statistical analyses were either performed using GraphPad Prism 7.0 or JMP 13.0

## Results

### ALX148 binds CD47 with enhanced affinity and prevents its interaction with wild-type SIRPα

**Fig 1:**
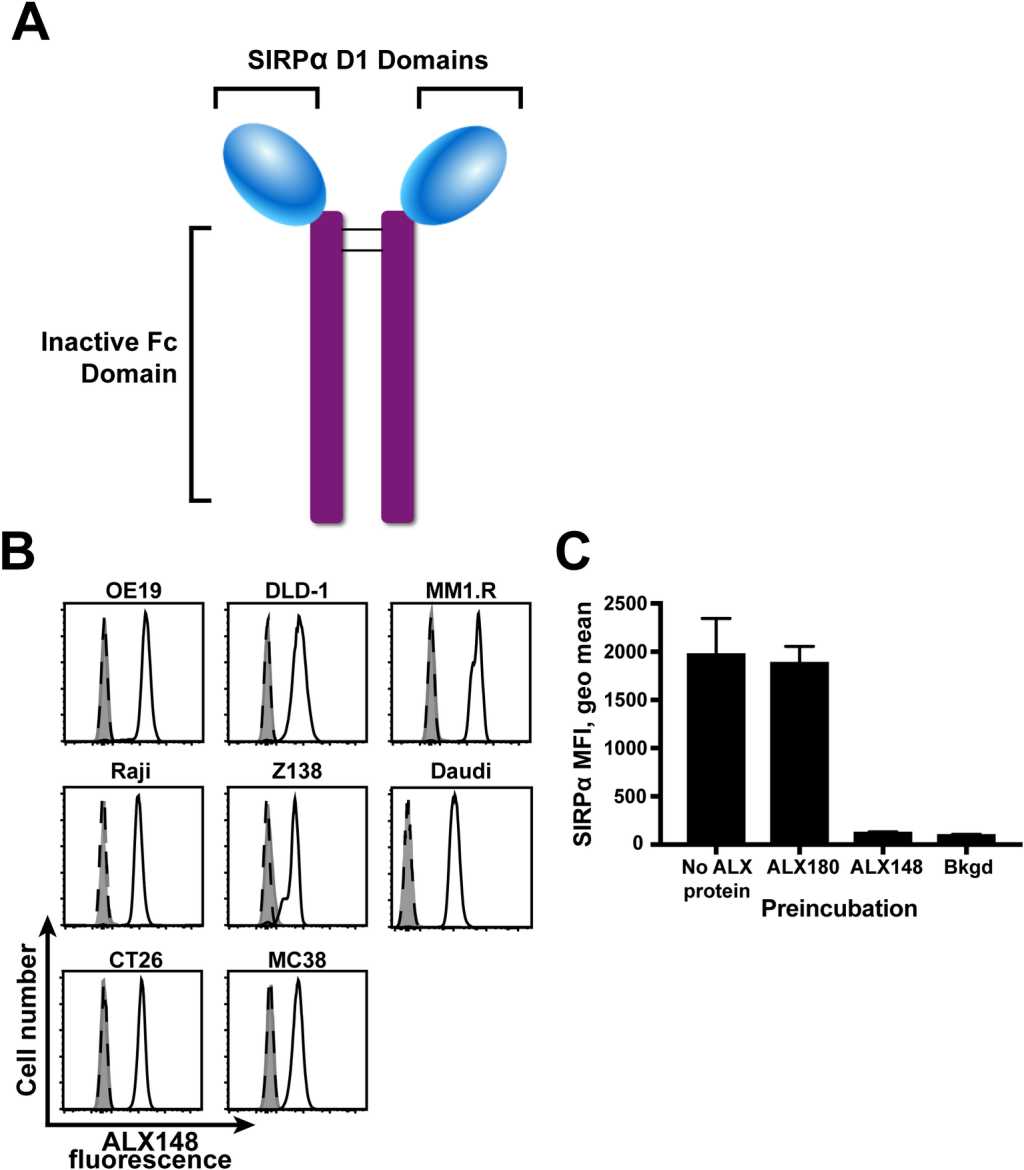
ALX148 binds human CD47 and prevents its interaction with SIRPα. (A) Structure of ALX148 molecule. Lines indicate disulfide bonds between ALX148 monomers. (B) Flow cytometry analysis of ALX148 binding (solid white histograms), negative control protein ALX180 binding (dashed histograms), or background fluorescence of unstained cells (filled histograms) for indicated human and mouse tumor cell lines. Cell number is normalized to mode. Results are representative of at least three independent experiments. (C) Flow cytometry analysis of wild-type SIRPα binding to Jurkat cells. Geometric mean fluorescence intensity is indicated on the y-axis. Cells were pre-incubated with proteins indicated on the x-axis prior to SIRPα incubation. Bkgd = background fluorescence in the absence of labeled wild-type SIRPα. Error bars indicate standard deviation of triplicates. Results are representative of three independent experiments.

ALX148 was constructed by genetically fusing an engineered version of the CD47-binding domain from human SIRPα to an inactive human IgG1 Fc domain (Fig 1A). ALX148 is a 78-kDa disulfide-linked homodimer that has no glycosylation sites. The affinity of ALX148 for CD47 of various species was determined using surface plasmon resonance (SPR) analysis. ALX148 bound human, cynomolgus monkey, mouse, and rat CD47 with high affinity at 25°C and 37°C (Table 1). ALX148’s affinity for human CD47 is 140 pM. The affinity of wild-type SIRPα for human CD47 is approximately 1 μM [44]. Therefore, ALX148 represents a dramatic increase in affinity for human CD47. The modifications to human SIRPα in ALX148 also dramatically increase the affinity for mouse CD47 to 14 nM, which allows the interrogation of ALX148 function in mice with an intact immune system.

**Table 1:** Apparent affinity of ALX148 for CD47 of various species.

Species	KD (M), 25°C	N	KD (M), 37°C	N
Human	1.4E-10	12	1.0E-09	12
Cynomolgous monkey	2.0E-10	16	9.6E-10	16
Mouse	1.4E-08	6	5.8E-08	4
Rat	8.1E-09	8	2.6E-08	8

N, number of independent experiments

To confirm that ALX148 binds CD47 expressed on the cell surface, a flow cytometry assay was used to measure the binding of fluorescently labeled ALX148 to cells (Fig 1B). As a control, the binding of ALX180, a variant of ALX148 engineered to be deficient for CD47 binding, was also evaluated. ALX148 bound all cell types tested, including human and mouse tumor cell lines. Fluorescence on cells incubated with ALX180 was the same as unstained cells, indicating that ALX180 does not bind cells under these conditions.

To determine if ALX148 binding to tumor cells prevents their interaction with wild-type SIRPα, the binding of SIRPα to tumor cell lines was evaluated using flow cytometry (Fig 1C). In this assay, binding of wild-type SIRPα to the Jurkat tumor cell line was measured with fluorescently labeled wild-type SIRPα fused to human IgG1 Fc. Binding of wild-type SIRPα to these cells was detected, with a 19-fold increase over background fluorescence at saturating concentrations. Preincubation with ALX148 caused a greater than 90 percent reduction in fluorescence, indicating that binding of the labeled wild-type SIRPα was blocked by ALX148. The negative control protein ALX180 did not decrease wild-type SIRPα binding. These results demonstrate that ALX148 binding to CD47 blocks the CD47-SIRPα interaction.

### ALX148 lacks effector function and binds the neonatal Fc receptor

To prevent the binding of ALX148 from having any deleterious consequences for normal CD47-expressing cells, amino acid substitutions were made within IgG1 Fc domain of ALX148 to eliminate interaction with both FcγRs and complement C1q protein [45–48]. To confirm the absence of ALX148 binding to FcγRs, SPR analysis was performed (Table 2). There was no detectable interaction between ALX148 and FcγRI, FcγRIIA, FcγRIIB/C, or FcγRIIIA. This was in contrast to ALX222, a positive control protein with wild-type IgG1 fused to a high affinity SIRPα domain. ALX222 interacted with all FcγRs tested as expected for an IgG1 molecule.

**Table 2:** Apparent affinity ALX148 for human Fcγ receptors.

Protein	FcγRIIIA KD (M)	FcγRIIA KD (M)	FcγRIIB/C KD (M)	FcγRI KD (M)
ALX148	NB	NB	NB	NB
ALX222	1.3E-07	4.2E-07	2.2E-06	2.9E-11

NB = No binding

The interaction with neonatal Fc receptor (FcRn) can impact the pharmacokinetic (PK) properties of Fc containing molecules. The substitutions in the Fc domain of ALX148 should not impact interaction with FcRn. SPR analysis confirmed binding of ALX148 to FcRn binding was unchanged, with a K_D_ of approximately 580 nM. This is equivalent to the 450 nM K_D_ observed for ALX222, the wild type IgG1 positive control (S1 Table).

All interaction with recombinant soluble FcγRs has been eliminated from ALX148. To confirm that ALX148 has no FcγR-mediated effector activity upon cells, a cell based antibody-dependent cellular cytotoxicity (ADCC) assay was used (Figure A in S1 Fig). In this assay, Fc binding induces signaling through FcγRIIIa, causing effector cells to express a luciferase reporter protein. Upon binding to CD47-positive Raji cells, ALX148 failed to induce luciferase expression, similar to a negative control IgG1 antibody that does not bind Raji cells. In contrast, the positive controls ALX222, which binds CD47, and rituximab, which binds CD20, induced robust luciferase expression due to their wild-type Fcs. These results demonstrate that although ALX148 binds CD47-positive cells, it lacks effector function and does not activate ADCC pathways.

To evaluate binding of ALX148 to complement C1q protein, an ELISA assay for binding of soluble complement C1q to immobilized proteins was performed (Figure B in S1 Fig). No complement C1q binding was observed for ALX148 or the negative control protein ALX217, which contains an IgG4 Fc that has no complement C1q binding activity [51]. In contrast, the signal for ALX222 was approximately 7-fold over background at the maximum concentration, indicating potent binding. These results demonstrate that binding to complement C1q has been removed from ALX148.

### ALX148 does not affect hematologic parameters in vitro or in vivo

**Fig 2:**
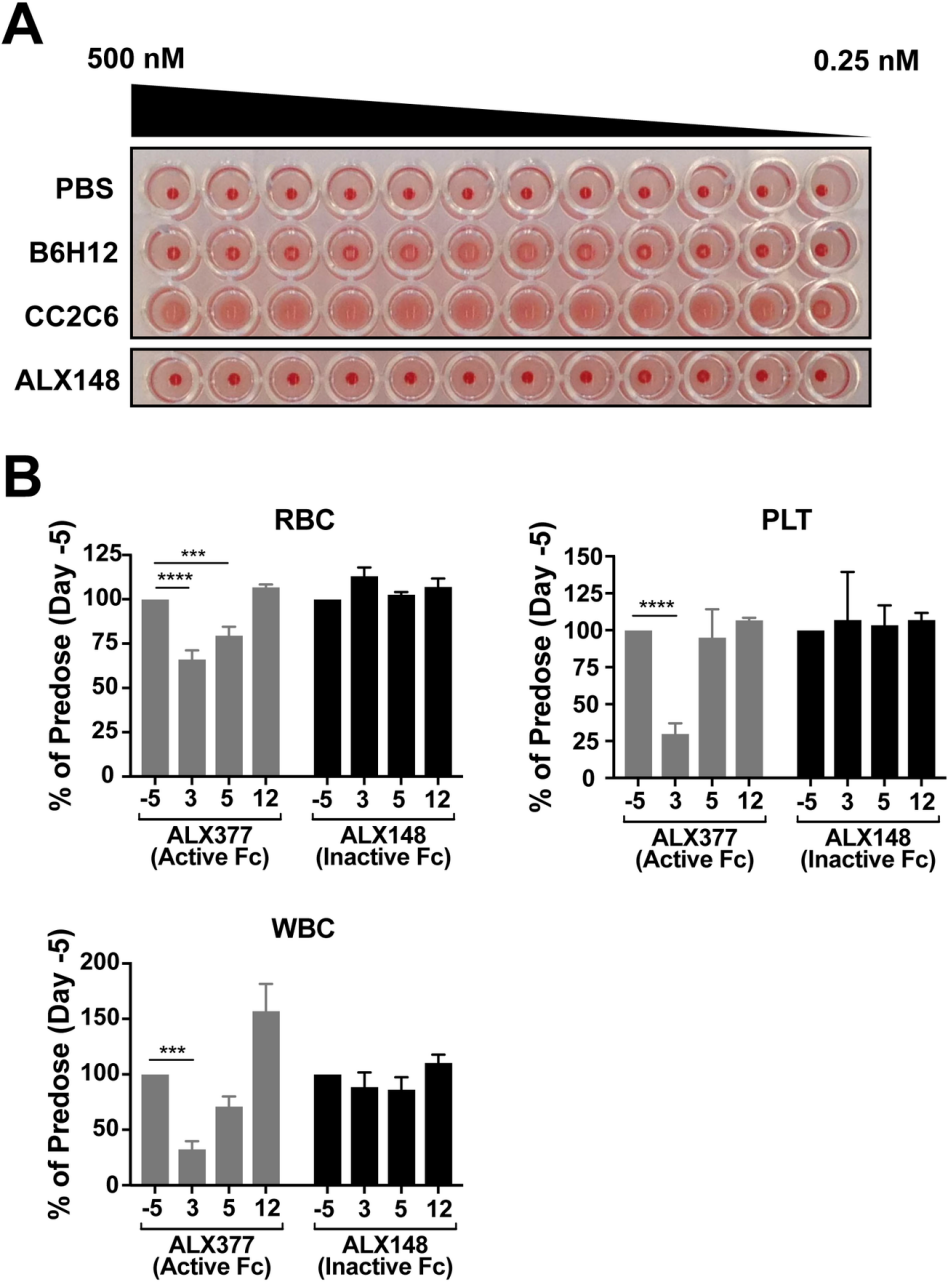
ALX148 does not affect hematologic parameters *in vitro* or *in vivo*. (A) Hemagglutination of human erythrocytes incubated with titrations of ALX148, the indicated antibodies against CD47, or PBS. Results shown are from the same experiment and are representative of three independent experiments. (B) CD-1 mice were administered 30mg/kg ALX377 or ALX148 intravenously. Each time point consists of 4–5 mice. RBC, platelet (PLT) and WBC counts for each mouse were normalized to their predose values. The mean normalized values for all mice at each time point and standard error mean (SEM) are indicated. ****p<0.0001, ***p<0.001. Statistics were performed using One-Way ANOVA, Tukey-Kramer.

Because CD47 is expressed on erythrocytes, molecules that bind CD47 could potentially cause hemagglutination. ALX148, however, did not induce hemagglutination at any concentration tested in *in vitro* assays with human erythrocytes. The anti-CD47 antibodies B6H12 and CC2C6 were used as positive controls, and these proteins induced hemagglutination as expected (Fig 2A).

The ADCC and C1q binding assays described above demonstrate that ALX148 lacks Fc effector function *in vitro*. To confirm the absence of effector function in an animal model, hematological parameters were evaluated in mice after administration of ALX148 or ALX377, a control protein with the identical SIRPα domain fused to a wild-type IgG1 Fc. This experiment was feasible due to the high affinity of the ALX148 SIRPα domain for mouse CD47 (Table 1), which confers robust binding to mouse cells (Fig 1B). Hematology for mice administered 30 mg/kg ALX148 showed levels of red blood cells (RBC), platelets, and white blood cells (WBC: lymphocytes, monocytes, and granulocytes) that were similar to the predose baseline (Fig 2B). In contrast, 30 mg/kg ALX377 resulted in 34% RBC, 70% platelet and 67% WBC reduction 3 days post dosing as compared to baseline (p<0.0001, p<0.0001 and p<0.001 respectively). By day 12, all levels in the 30mg/kg ALX377 group returned to baseline. These results demonstrate that the removal of effector function from ALX148 eliminates any adverse effects on normal blood cells.

### ALX148 enhances antibody-dependent phagocytosis of tumor cell lines

**Fig 3:**
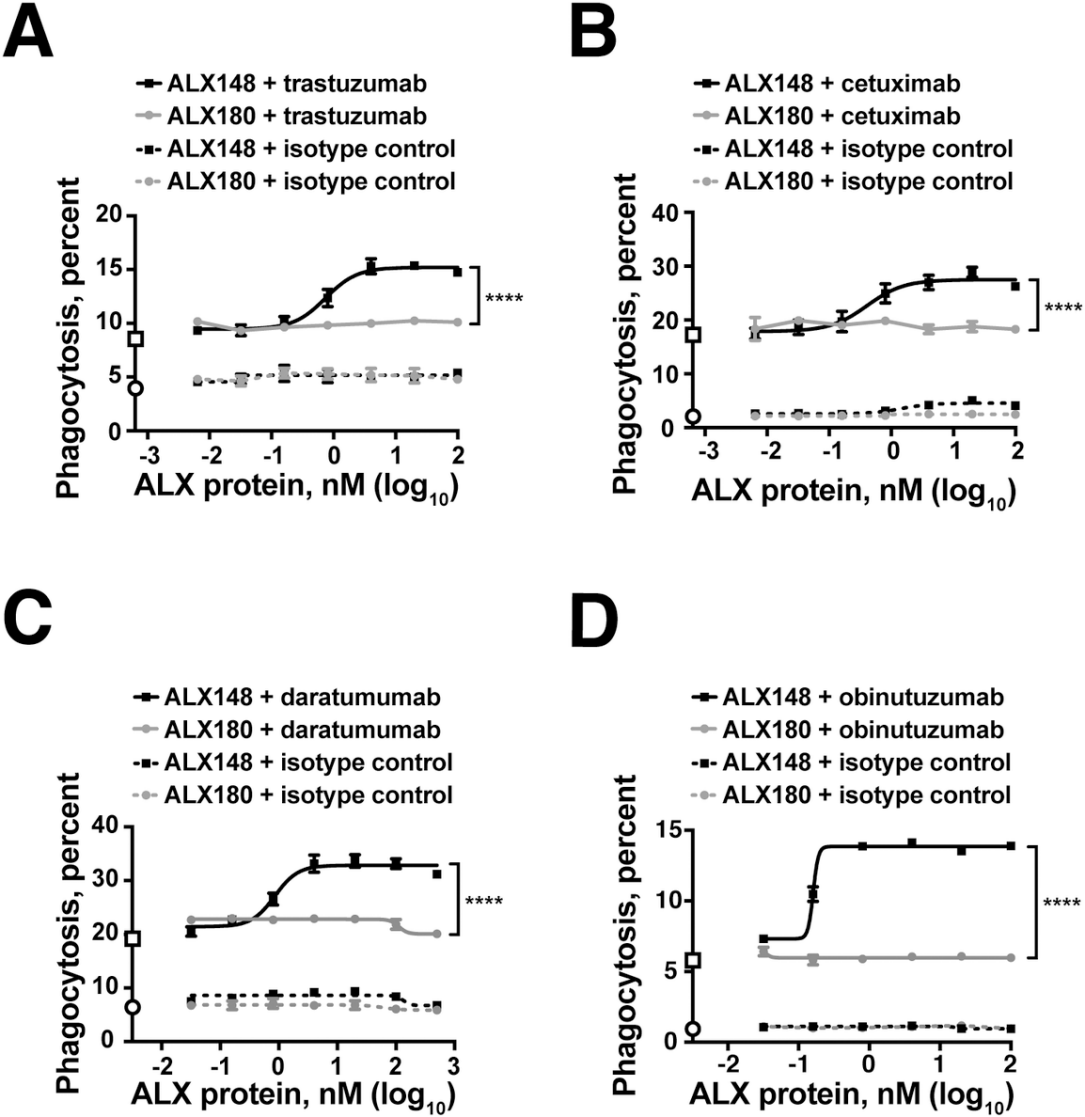
ALX148 enhances antibody-dependent cellular phagocytosis. *In vitro* phagocytosis experiments with human monocyte-derived macrophages and tumor cell lines. Percent of macrophages that engulfed tumor cells is indicated on the y-axis. On y-axis, open circle indicates background phagocytosis observed with isotype control antibody and open square indicates cells treated with antitumor antibody alone. Antitumor antibody concentrations were constant and ALX proteins were titrated. Cells were treated with antitumor antibody plus ALX148 (solid black line) or antitumor antibody plus ALX180 (solid grey line). Cells were then treated with control antibody plus ALX148 (dotted black line) or control antibody plus ALX180 (dotted grey line). Tumor cell line and antibody combinations were (A) OE19 cells and trastuzumab, (B) DLD-1 GFP Luciferase cells and cetuximab, (C) MM1.R cells and daratumumab, and (D) Daudi cells and obinutuzumab. Error bars represent standard deviation of triplicates. For all antitumor antibodies tested, 100 nM ALX148 significantly enhanced antibody-dependent cellular phagocytosis (****p<0.0001 one-way ANOVA, Tukey-Kramer) compared to antibody alone. Results are representative of at least three independent experiments.

The elimination of tumor cells by macrophage phagocytosis is an important component of the innate antitumor immune response. In antibody-dependent cellular phagocytosis (ADCP), naturally occurring or therapeutically administered antibodies initiate this process by binding target cells and engaging macrophage FcγRs. CD47 blockade with ALX148 could potentially enhance this process by preventing the interaction of CD47 with SIRPα on macrophages. To determine the effect of ALX148 on ADCP, a flow cytometry-based *in vitro* phagocytosis assay with human monocyte-derived macrophages was used to evaluate the combination of ALX148 with multiple therapeutic antitumor antibodies. Immunofluorcence images of macrophages confirm the phagocytosis of whole CFSE-labeled target cells inside macrophages (Figures in S2 Fig). The antitumor antibodies trastuzumab, cetuximab, daratumumab, and obinutuzumab had ADCP activity, causing phagocytosis of OE19, DLD-1, MM1.R, and Daudi cells, respectively (Fig 3). In each case, ALX148 enhanced the phagocytosis activity of the antitumor antibody in a dose-dependent manner. ALX180, which is deficient for CD47 binding, conferred no increase in phagocytosis over the levels observed with antitumor antibody alone. ALX148 does not induce phagocytosis in the absence of an antitumor antibody, consistent with the elimination of effector function from the ALX148 Fc domain. These results show that ALX148 enhances the ADCP activity of multiple antitumor antibodies and that CD47 binding is essential for this enhancement.

### ALX148 enhances antitumor therapy in vivo

**Fig 4:**
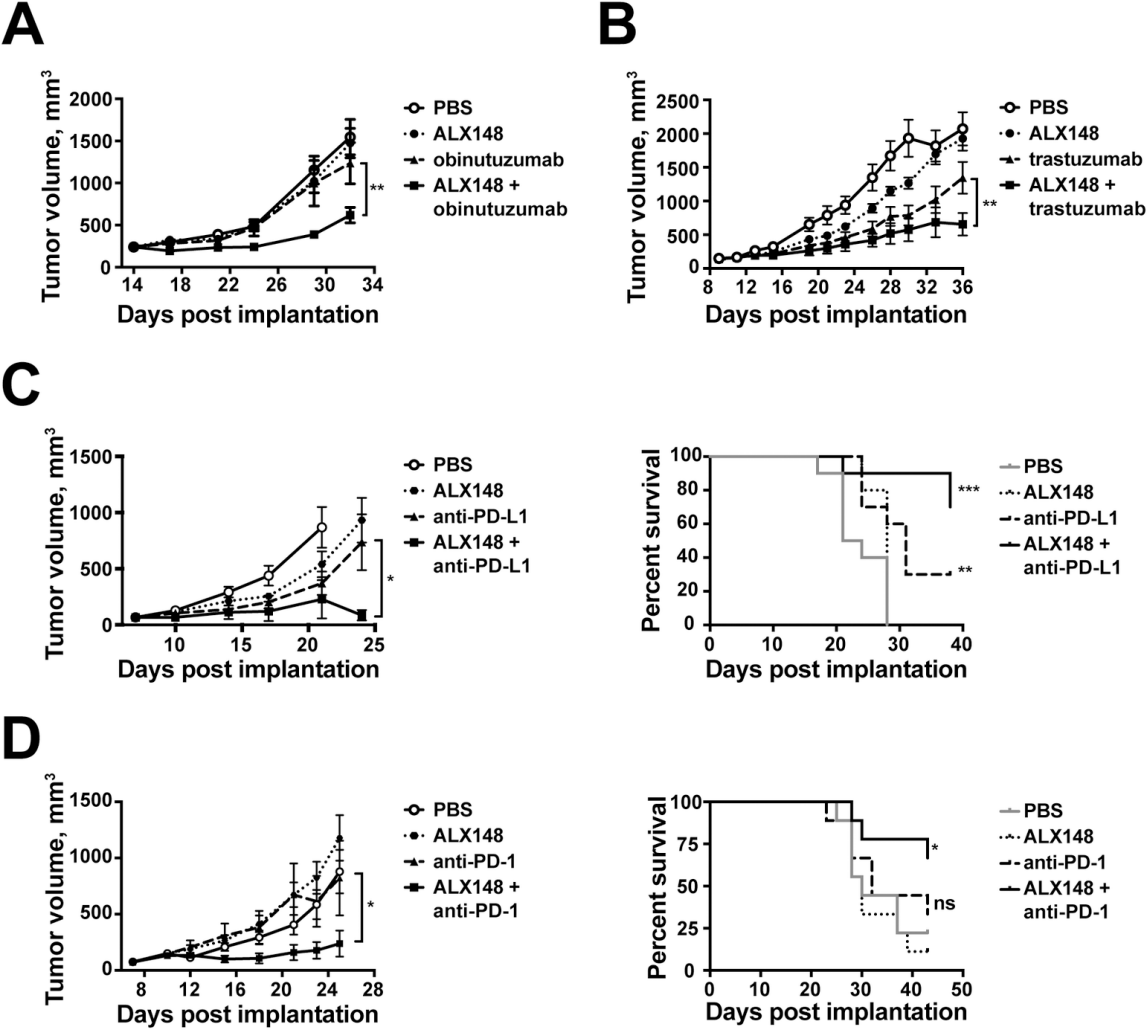
ALX148 enhances antitumor therapy *in vivo*. (A) Z138 B-cell mantle cell lymphoma and (B) OE19 gastric tumor cells were implanted subcutaneously on the right flank of NOD-SCID mice. Mice with established tumors (average of 240 mm^3^ for Z138 and 140 mm^3^ for OE19) were randomized and treated i.p. with vehicle, ALX148, obinutuzumab for Z138 or trastuzumab for OE19 or combination of ALX148 and anti-cancer antibody. Graphs show mean tumor growth ± SEM of n = 10 mice. ALX148 in combination with obinutuzumab or trastuzumab showed significant inhibition of tumor growth as compared to obinutuzumab or trastuzumab monotherapy, **p<0.01, day 32 and **p<0.01, day 36 (two-tailed student’s t-test), respectively. (C) MC38 and (D) CT26 colon carcinoma cells were implanted subcutaneously on the right flanks of C57BL/6 and BABL/C mice, respectively. When tumors reached an average of 70–75 mm^3^, mice were randomized into groups and treated. (C) Mice bearing MC38 tumors were treated with PBS, ALX148, anti-PD-L1 or ALX148 + anti-PD-L1. Graphs show tumor growth ± SEM of n = 10 mice (left panel) and survival curves (right panel). ALX148 + anti-PD-L1 shows significant inhibition of tumor growth as compared to anti-PD-L1 monotherapy, (*p< 0.05, day 24 two-tail student’s t-test). Survival of ALX148 + anti-PD-L1 and anti-PD-L1 alone are significantly increased compared to PBS alone (***p<0.001 and **p<0.01, respectively, log-rank (Mantel-Cox) test). (D) Mice bearing CT26 tumors were treated with PBS, ALX148, anti-PD-1 or ALX148 + anti-PD-1. Graphs show tumor growth ± SEM of n = 9 mice (left panel) and survival curves (right panel). ALX148 + anti-PD-1 shows significant inhibition of tumor growth as compared to anti-PD-1 monotherapy, (*p< 0.05, day 25 two-tail student’s t-test) and significant increased survival as compared to PBS alone (*p<0.05, log-rank (Mantel-Cox) test). ns, not significant.

The ability of ALX148 to enhance the activity of targeted antitumor antibodies *in vivo* was assessed using mouse subcutaneous xenograft models of human tumors. In the Z138 B-cell mantle cell lymphoma model, therapeutic treatment with ALX148 in combination with obinutuzumab resulted in enhanced inhibition of tumor growth compared to mice treated with obinutuzumab or ALX148 alone (p<0.01) (Fig 4A). Similarly, in mice harboring OE19 gastroesophageal tumors, ALX148 in combination with trastuzumab significantly inhibited tumor growth as compared to mice treated with trastuzumab alone in a therapeutic treatment model (p<0.01) (Fig 4B). Lastly, the combination of ALX148 with rituximab significantly enhanced the inhibition of tumor growth compared to treatment with rituximab alone and increased survival as compared to PBS in Raji B-cell lymphoma tumors (p<0.001 and p<0.0001, respectively) (Figure A in S3 Fig). These results are consistent with previous studies in which the inhibition of CD47 enhanced the efficacy of rituximab against established Raji tumors [25, 32]. Thus, ALX148 mediates significant tumor growth inhibition and longer survival when used in combination with anti-cancer antibodies at concentrations with limited single agent efficacy.

One limitation of xenograft models is the requirement for an immunodeficient host, which prevents the full effect of ALX148 on the immune system from being addressed. Furthermore, these models may be unusually dependent upon the CD47- SIRPα interaction due to the high affinity of NOD-SCID SIRPα for human CD47. To assess whether ALX148 engages the adaptive arm of the immune system to potentiate antitumor activity, the efficacy of ALX148 was investigated as a monotherapy and in combination with checkpoint blockade in immunocompetent syngeneic tumor models. MC38 and CT26 tumor bearing mice were treated when tumors reached an average of 70–75 mm^3^. ALX148 alone showed minimal single agent activity when administered twice at 30 mg/kg, ten days apart. However, ALX148 in combination with anti-PD-L1 showed enhanced tumor growth inhibition as compared to anti-PD-L1 monotherapy in mice bearing established MC38 tumors (p<0.05) (Fig 4C). In CT26 tumor bearing mice, combination treatment with ALX148 and anti-PD-1 resulted in enhanced tumor growth inhibition as compared to anti-PD-1 treatment group (p<0.05) (Fig 4D). Furthermore, ALX148 extended the survival of both MC38 and CT26 tumor bearing mice when used in combination with anti-PD-1 or anti-PD-L1 (p<0.001 and p<0.05 as compared to PBS group). ALX148 was also tested in combination with an agonistic T-cell activator, anti-4-1BB, in CT26 tumor-bearing mice. This combination showed a significant enhancement of survival as compared to PBS (Figure B in S2 Fig). These data in immunocompetent mice show that ALX148 enhances antitumor activity of immunotherapeutic molecules that directly enhance T cell-mediated adaptive immunity.

### ALX148 reduces myeloid-driven immune suppression

**Fig 5:**
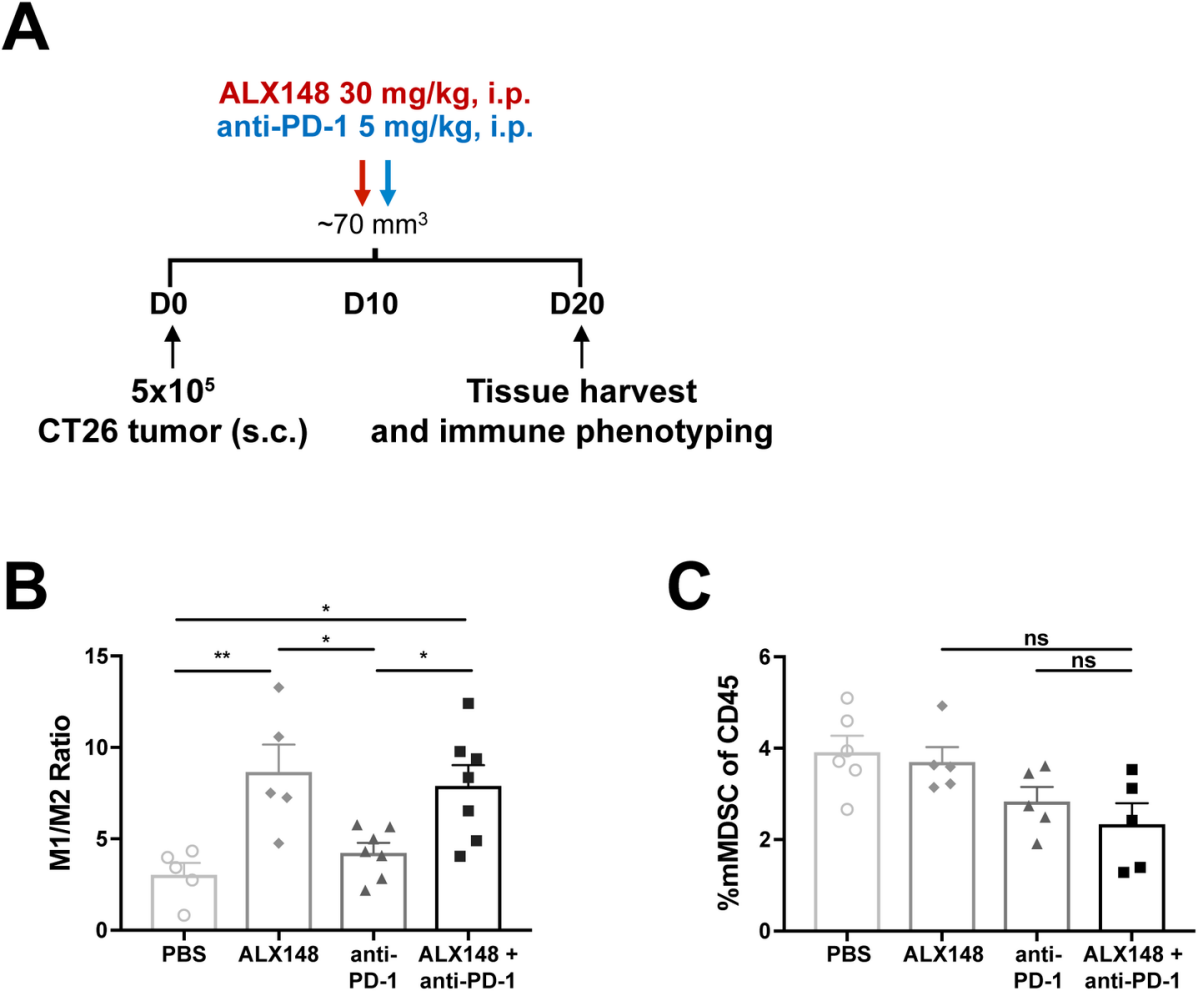
ALX148 reduces myeloid-driven immune suppression in tumor. (A) Schematic of dosing schedule and tissue harvest for immune phenotyping. CT26 colon carcinoma cells were implanted subcutaneously in BALB/c mice and treated on day 10 post-implantation with a single dose of PBS, ALX148, anti-PD-1 or ALX148 + anti-PD-1. 10 days post-treatment, spleen and tumor were harvested for immune phenotyping. (B) Ratio of M1/M2 TAMs in tumor. M1 is defined as CD45^+^CD11b^+^CD38^+^EGR2^-^ and M2 is defined as CD45^+^CD11b^+^CD38^-^EGR2^+^. Results are representative of two independent experiments of n = 5–7 mice/group. (C) Percent mMDSC in tumor. mMDSC is defined as CD45^+^CD11b^+^Ly6C^hi^MHCII^-^. Results are representative of two independent experiments of n = 5–6 mice/group. *p<0.05, **p<0.01. Statistics were performed using One-Way ANOVA, Tukey-Kramer.

Given the established effect of CD47 blockade on macrophage activity, we examined the effect of ALX148 on myeloid cells within the tumor microenvironment. In the tumor, tumor-associated macrophages (TAMs) have pro-inflammatory (M1) and suppressive (M2) activities [52, 53]. To identify the effect of ALX148 on TAM populations, flow cytometric analysis was conducted on CT26 tumors isolated 10 days after administration of either ALX148 (30 mg/kg), anti-PD-1 (5 mg/kg), a combination of ALX148 and anti-PD-1, or vehicle control (Fig 5A). ALX148 alone resulted in a nearly 3-fold increase in the ratio of M1 to M2 macrophages and the combination of ALX148 and anti-PD-1 resulted in a 2.5-fold increase in the ratio compared to the vehicle control (p<0.01 and p<0.05, respectively) (Fig 5B). Anti-PD-1 alone had no effect on TAMs. In addition, there was a 1.7-fold decrease in monocytic myeloid-derived suppressor cells (mMDSC) compared to vehicle control when ALX148 was combined with anti-PD-1 (Fig 5C). These results suggest that, through CD47 blockade, a single dose of ALX148 can change the composition of TAM populations toward a more anti-tumor, less suppressive phenotype and reduce mMDSCs within the tumor microenvironment.

### ALX148 induces dendritic cell activation

**Fig 6:**
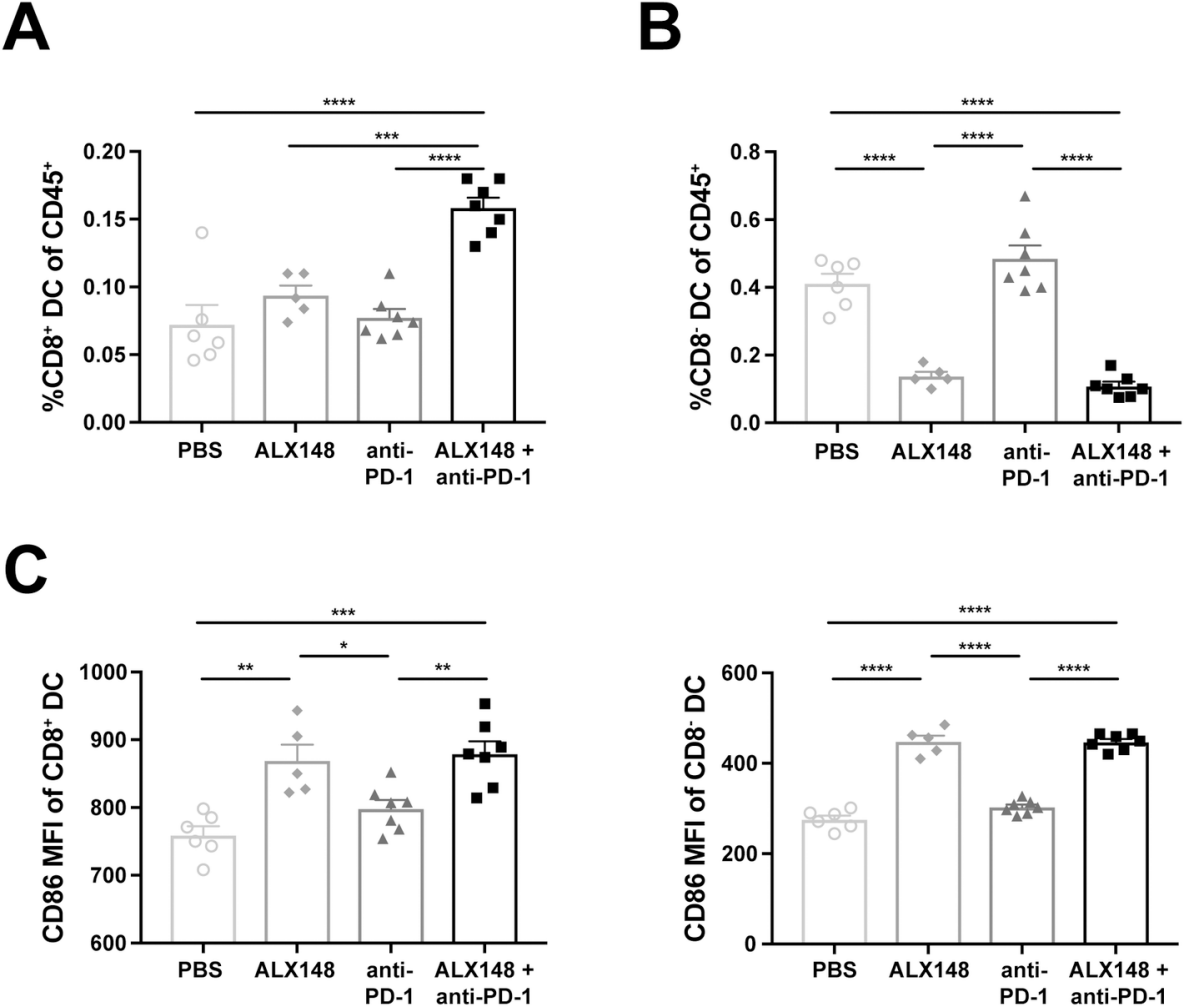
ALX148 induces splenic dendritic cell activation. Spleen of CT26 tumor-bearing mice, 10 days post single-dose of PBS, ALX148, anti-PD1 or ALX148 in combination with one dose of anti-PD1. (A) Percent splenic CD8^+^ DC of CD45^+^ cells. (B) Percent splenic CD8^-^ DCs of CD45^+^ cells. (C) Geometric MFI of CD86^+^ splenic CD8^+^DCs (left panel) and CD8^-^DCs (right panel). Results are representative of at least two independent experiments of n = 5–7 mice/group. *p<0.05, **p<0.01, ***p<0.001 and ****p<0.0001. Statistics were performed using One-Way ANOVA, Tukey-Kramer.

DCs and macrophages are highly effective at phagocytosis and antigen presentation, establishing them as potent regulators of innate and adaptive immune responses. Because DCs play a particularly important role in naïve T cell activation and initiation of antigen-specific immune responses [54, 55], we investigated the effect of ALX148 on these cells in tumor-bearing mice. A single combination dose of ALX148 and anti-PD-1 significantly increased the percentage of CD8^+^ DCs in the spleen 2.2-fold over vehicle, while neither agent affected DCs on its own (Fig 6A). Interestingly, ALX148 alone or in combination with anti-PD-1 also leads to a nearly 4-fold decrease in the percentage of CD8^-^ DCs, while anti-PD-1 alone had no effect (Fig 6B). A similar decrease in CD8^-^ DCs was previously reported in non-tumor bearing mice treated with antibodies blocking the CD47-SIRPα interaction [37]. Furthermore, ALX148 mediated an increase in DC activation, shown by upregulation of the activation marker CD86 in both CD8^+^ and CD8^-^ DCs, while anti-PD-1 showed no such effect (Fig 6C). These studies demonstrate that ALX148 induces DC activation and a decrease in CD8^-^ DCs as a single agent, while an increase in CD8^+^ DCs is evident when ALX148 is combined with anti-PD-1.

### ALX148 bridges innate and adaptive immune responses

**Fig 7:**
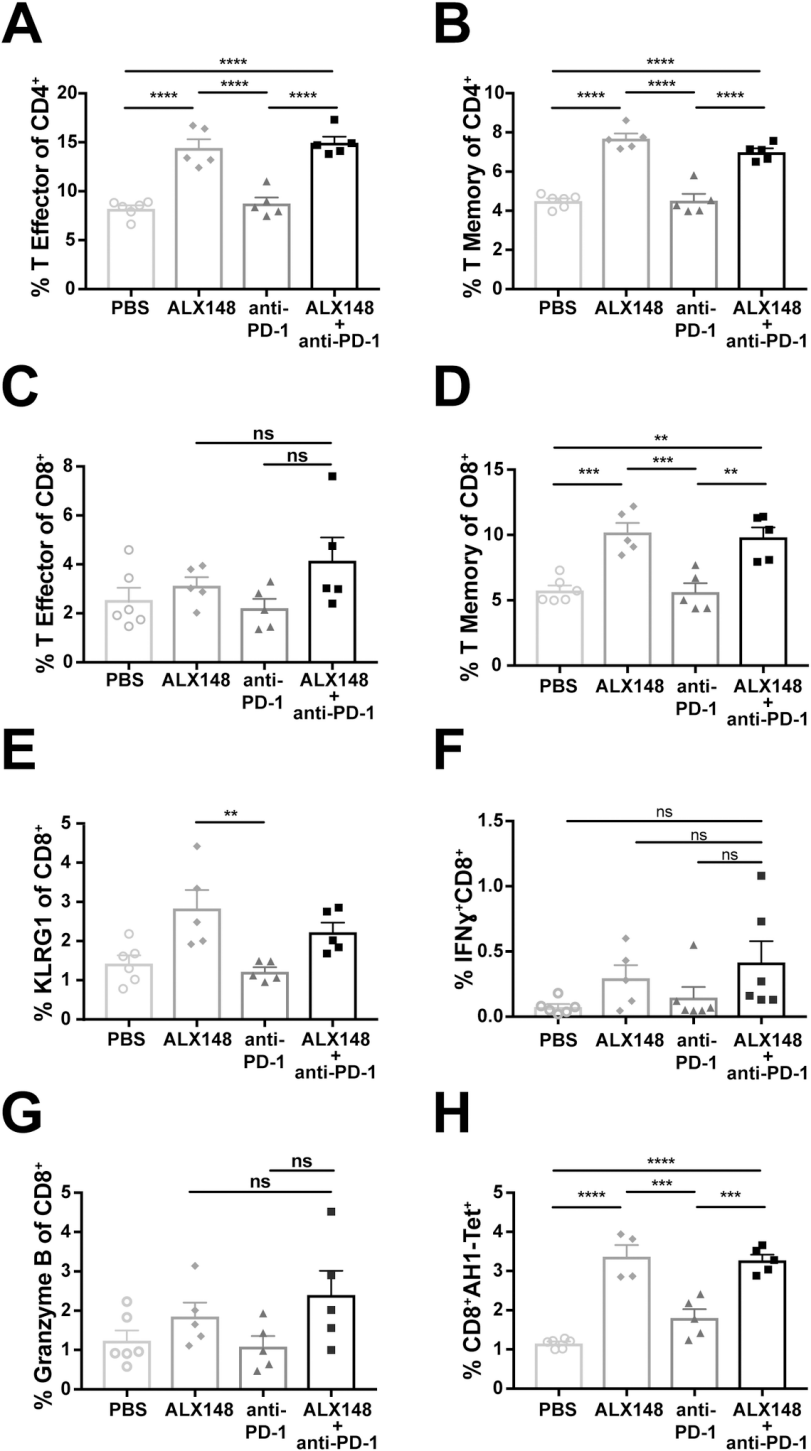
ALX148 activates adaptive immune response in the spleen. Spleen of CT26 tumor-bearing mice, 10 days post single-dose of PBS, ALX148, anti-PD-1 or ALX148 in combination with one dose of anti-PD-1. (A-B) Percent effector memory (CD44^+^CD62L^-^) and central memory (CD44^+^CD62L^+^) CD4^+^ T cells. (C-D) Percent effector memory and central memory CD8^+^ T cells. (E) KLRG1 expression on CD8^+^ T cells. (F) Percent intracellular IFNγ expressing CD8^+^ T cells following *ex vivo* stimulation of splenocytes with PMA/ionomycin and Golgi-Stop for 4 hours. (G) Percent intracellular granzyme B expressing CD8^+^ T cells. (H) Percent CD8^+^AH1-tet^+^ T cells following *ex vivo* stimulation of splenocytes with 10 μg/mL AH1 peptide for 4 hours. Results are representative of 1–3 independent experiments of n = 4–6 mice/group. **p<0.01, ***p<0.001 and ****p<0.0001. Statistics were performed using One-Way ANOVA, Tukey-Kramer.

Because of the role played by DCs in T cell activation, we reasoned that DC activation by ALX148 might result in subsequent enhancement of T cell responses. To investigate this hypothesis, CD4^+^ and CD8^+^ T cell populations were evaluated in the spleens of tumor bearing mice that received ALX148 alone or in combination with anti-PD-1, which activates adaptive T cell responses by inhibition of the PD-1 checkpoint [56]. As predicted, ALX148 alone or in combination with anti-PD-1 mediated robust increases over vehicle control in splenic effector memory (CD44^+^CD62L^-^) and central memory (CD44^+^CD62L^+^) CD4^+^ T cell populations (p<0.0001 for both subsets). Anti-PD-1 had no such effect as a single agent (Fig 7A and 7B). Similarly, CD8^+^ T cells revealed increased effector memory phenotype (Fig 7C) and a significant concomitant increase in central memory CD8^+^ T cells (Fig 7D) in the presence of ALX148 alone or in combination with anti-PD-1 compared to the vehicle control (p<0.001 and p<0.01, respectively). This enhanced effector function was further supported by increased KLRG1 expression (Fig 7E). In addition, the cytotoxic potential of these CD8^+^ T cells was confirmed by enhanced IFNγ and Granzyme B expression upon treatment with ALX148 alone or in combination with anti-PD-1 (Fig 7F and 7G). CT26 tumor cells express the endogenous H2-L^d^ restricted antigen, AH-1 [57–59]. To identify tumor-specific immune responses, AH1-specific CD8^+^ T cells were interrogated with the AH1 peptide or its corresponding H2-L^d^ restricted tetramer. As expected, AH1-specific CD8^+^ T cells were limited to CT26 tumor-bearing mice and were absent both from mice harboring MC38 tumors, which do not present the AH1 peptide, and from tumor-free BALB/c or C57BL/6 mice that were used as the negative control (Figures in S4 Fig). In CT26 tumor-bearing mice, ALX148 treatment significantly enhanced the induction of AH1^+^CD8^+^ T cells compared to the vehicle control (Fig 7H, p<0.0001) and not in mice harboring MC38 tumors regardless of treatment (Figure B in S4 Fig). These data demonstrate that ALX148 can enhance splenic memory and cytotoxic T cell phenotypes both as a single agent and in combination with anti-PD-1.

**Fig 8:**
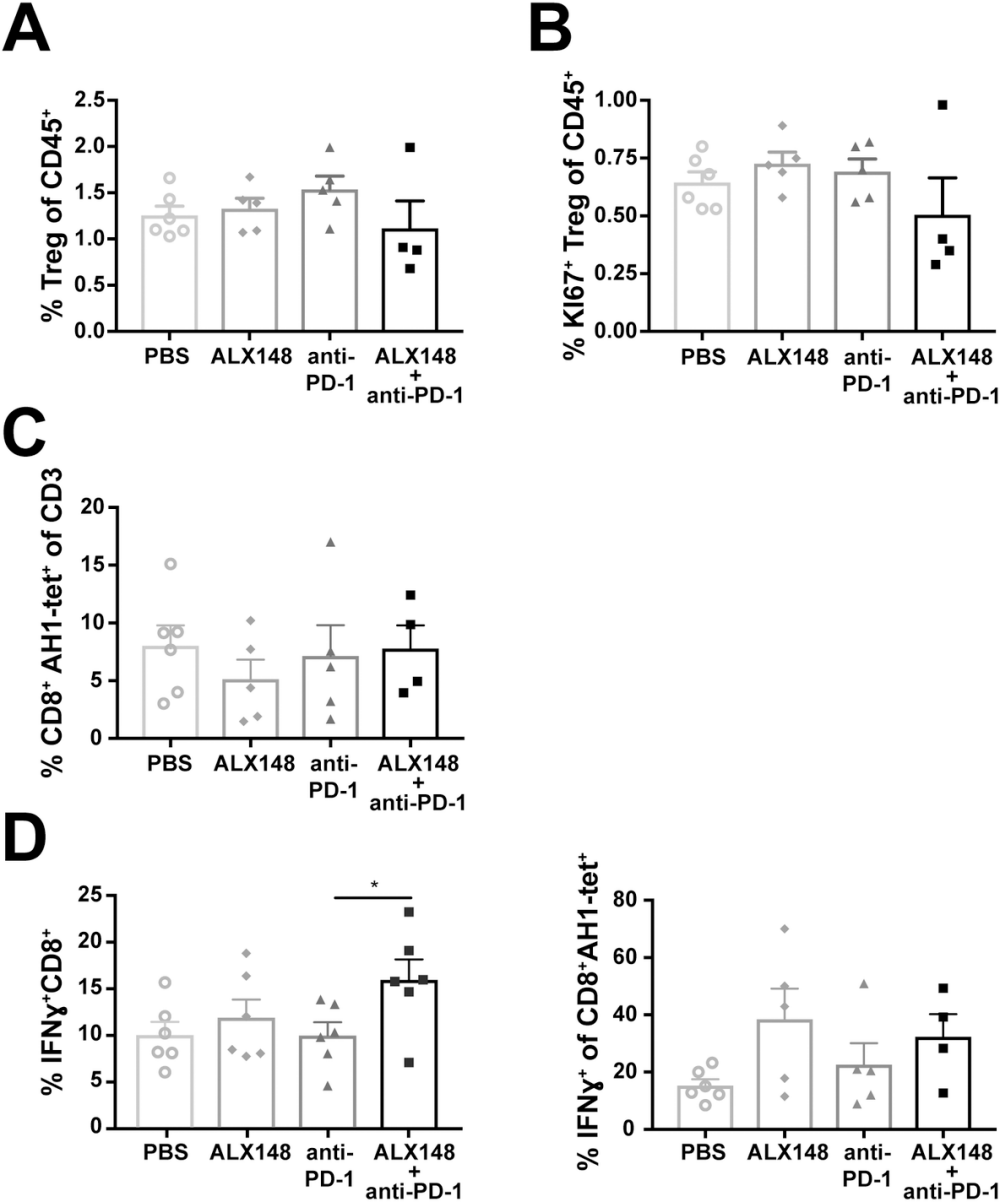
Combination of ALX148 and anti-PD-1 reduces suppressive tumor microenvironment and activates adaptive immune response in CT26 tumor. (A) Percent FOXP3^+^CD25^+^ in tumor. (B) Percent Ki67^+^ Tregs in tumor. (C) Percent AH1-tet^+^CD8^+^ T cells. Following antibody treatment, *ex vivo* tumor-derived single cell suspension stimulation with AH1 peptide at 10 μg/mL. (D) Percent intracellular IFNγ expressing CD8^+^ T cells (left panel) and percent IFNγ^+^ of AH1-tet^+^CD8^+^ T cells (right panel). Following antibody treatment, *ex vivo* tumor-derived single cell suspension were stimulated in the presence of PMA/ionomycin or AH1 peptide at 10 μg/mL. Results are representative of 1–3 independent experiments of n = 5–6 mice/group.

To provide evidence for a localized antitumor immune response, the impact on tumor infiltrating T-lymphocytes (TILs) was assessed. In general, the effects on TILs were less robust than those observed in the spleen. Interestingly, the combination treatment of ALX148 and anti-PD-1 showed a trend towards reduced CD4^*+*^Treg infiltrates and decreased proliferative potential as evidenced by reduced Ki67 staining (Fig 8A and 8B). No changes in either effector memory (not shown) or CT26 antigen-specific AH1^+^ CD8^*+*^ T cells (Fig 8C) were observed across all treated groups. However, when TILs were stimulated *ex vivo* with PMA and ionomycin, a significant increase in IFNγ-expressing CD8^*+*^T cells was observed in mice that received combination treatment (p<0.05 compared to anti-PD-1 only) (Fig 8D, left panel). A similar IFNγ expression phenotype was seen in tumor infiltrating CD8^+^ AH1-tet^+^ T cells from mice treated with ALX148 and in combination with anti-PD-1. However, this trend was not statistically significant. (Fig 8D, right panel).

**Fig 9:**
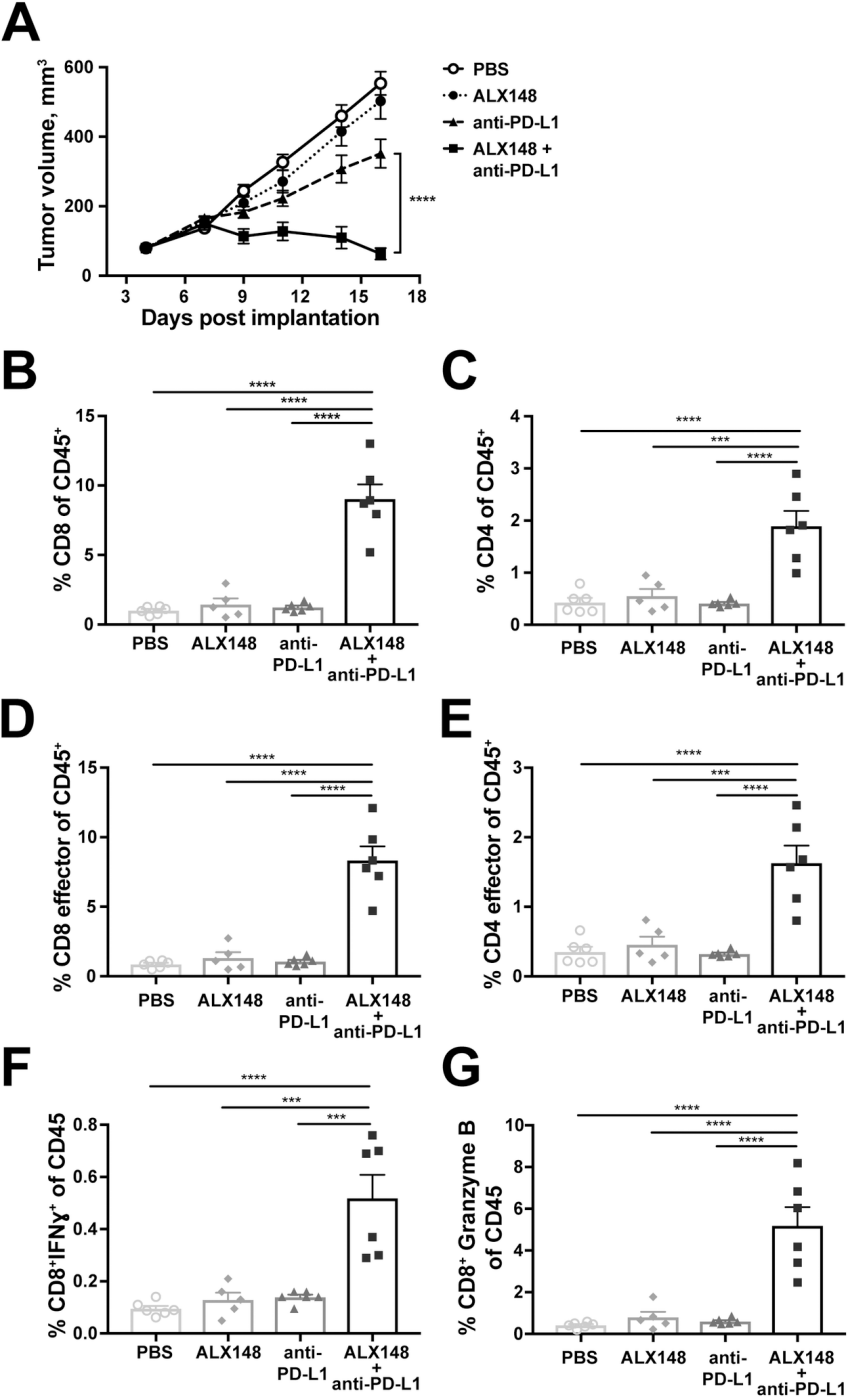
Combination of ALX148 and anti-PD-L1 enhances adaptive immune response in MC38 tumor model. (A) MC38 colon carcinoma cells were implanted subcutaneously in C57BL/6 mice and treatment was initiated four days post-implantation. Treatment consisted of PBS, ALX148, anti-PD-L1 or ALX148 + anti-PD-L1. ALX148 was dosed twice at 30 mg/kg, ten days agart and anti-PD-L1 was dosed twice, one per week. On day 17 post-tumor challenge, tumors were harvested for immune phenotyping. (B-C) Percent tumor infiltrating CD8^+^ and CD4^+^ T cells ^+^ (D-E) Percent effector memory (CD44^+^CD62L^-^) CD8 and CD4 in tumor (F) Percent IFNγ expression in CD8+ T cells (G) Percent Granzyme B expression in CD8+ T cells. All values are reported as percent of CD45 with 5–6 mice/group. ***p<0.001 and ****p<0.0001. Statistics were performed using One-Way ANOVA, Tukey-Kramer.

A similar analysis of TILs was performed in MC38 tumor bearing mice treated with two doses of either ALX148 alone, anti-PD-L1 alone, or both. ALX148 in combination with anti-PD-L1 shows significant tumor growth inhibition as compared to anti-PD-L1 treated group (p<0.0001) (Fig 9A). Two days post last treatment of ALX148, a significant increase in tumor infiltrating CD8^+^ (p<0.0001) and CD4^+^ T cells (p<0.0001) (Fig 9B and 9C) was seen in mice receiving combination treatment compared to vehicle control. Further profiling of these T cells revealed an enhanced effector memory phenotype (CD44^+^CD62L^-^) (p<0.0001, both CD8 and CD4) (Fig 9D and 9E) with concomitant increase in effector function and cytotoxic potential as evidenced by increased IFNγ (p<0.0001), and granzyme B (p<0.0001) within TILs of mice receiving both ALX148 and anti-PD-L1 compared to vehicle control (Fig 9F and 9G). In the MC38 model, ALX148 combination therapy resulted in a more robust immune response and greater tumor growth inhibition than in the CT26 model. This association indicates that enhancement of the adaptive immune response by ALX148 may drive increased antitumor efficacy.

Collectively, these data show that ALX148 strongly activates the adaptive immune response in an antigen-specific manner within the spleen, and that such activation may lead to an increased antitumor response.

### Higher doses of ALX148 dramatically increase serum titer and lead to greater tumor penetration

**Fig 10:**
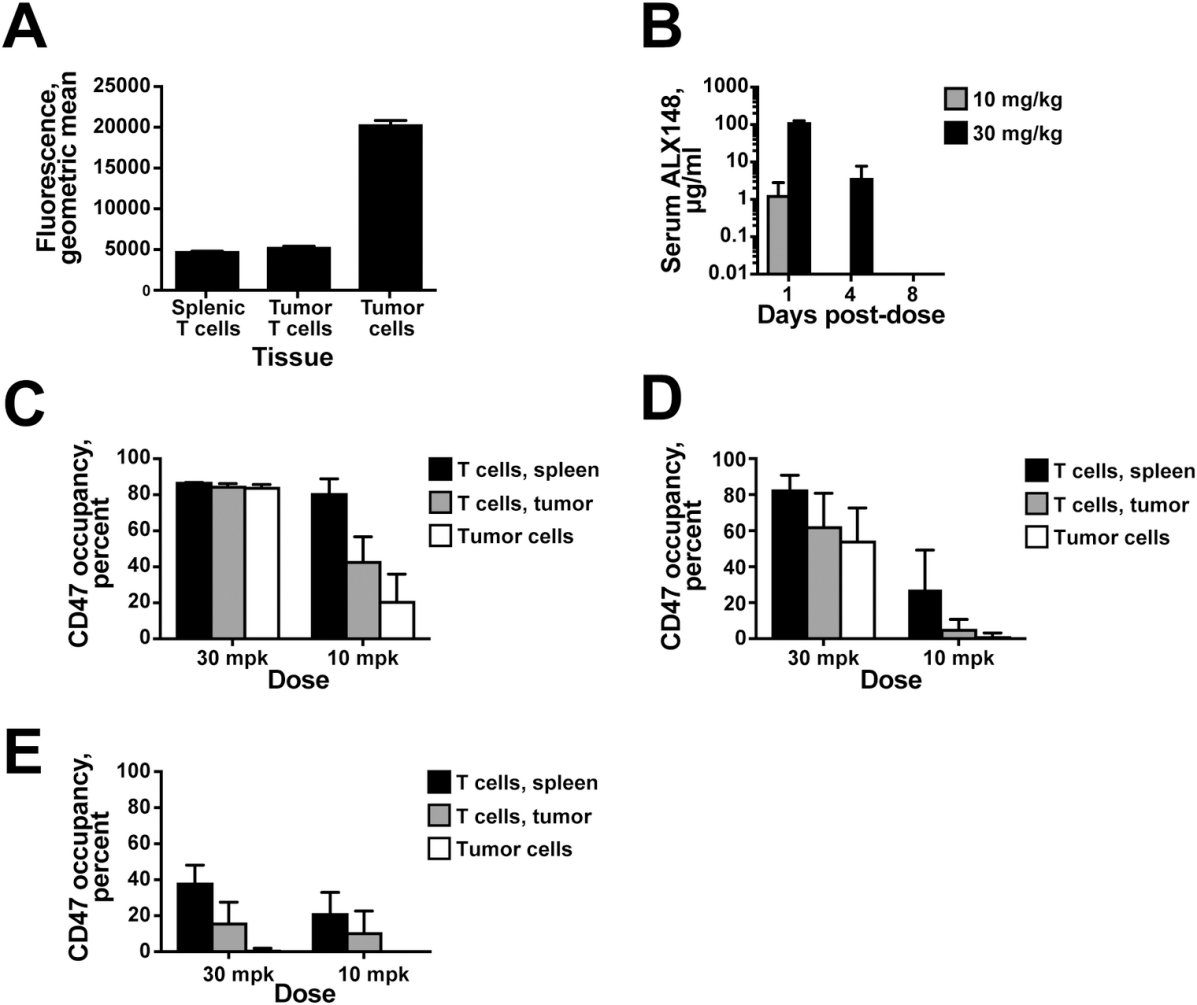
Higher doses of ALX148 saturate CD47 antigen sink and drive tumor penetration. (A) ALX148 binding to the indicated cells from naïve mice. (B) Serum level of ALX148 at the indicated times after administration of 10 mg/kg (grey column) or 30 mg/kg (black column). (C-E) CD47 occupancy in splenic CD4^+^ T cells (black columns), tumor-infiltrating CD4^+^ T cells (grey columns), or tumor cells (open columns) (C) one, (D) four, or (E) eight days after administration of the indicated dose. Mean of five mice is shown and error bars represent standard deviation. Results are representative of two independent experiments.

To better understand the relationship between ALX148 PK and pharmacodynamics in the tumor, ALX148 serum concentration and CD47 occupancy were analyzed in mice harboring syngeneic tumors. In mice that did not receive ALX148, staining with labeled ALX148 revealed greater CD47 expression on tumor cells than on CD4^+^ cells from the spleen or tumor (Fig 10A). In mice that received ALX148, a three-fold increase in dose from 10 mg/kg to 30 mg/kg resulted in an approximately 100-fold increase in the ALX148 serum level at 24 hours (Fig 10B). This nonlinear increase in ALX148 exposure is consistent with a saturable CD47 antigen sink. At the four day time point, serum ALX148 was only detected at the 30 mg/kg dose, and at the eight day time point serum ALX148 was undetectable at either dose.

Measurement of CD47 occupancy demonstrated dose-dependent effects on both tissue distribution and persistence. The 10 mg/kg dose resulted in 80 percent CD47 occupancy on splenic T cells twenty-four hours post-dose, while only 21 and 43 percent occupancy was observed for tumor cells and T cells, respectively, in the tumor compartment. In contrast, the 30 mg/kg dose achieved near maximal occupancy on all cell types assayed, with 87, 85, and 84 percent occupancy detected on splenic CD4^+^ T cells, tumor cells, and tumor-infiltrating CD4^+^ T cells, respectively (Fig 10C). Analysis of CD47 occupancy four days (Fig 10D) and eight days (Fig 10E) after dosing showed that CD47 occupancy persisted longer after the 30 mg/kg dose than the 10 mg/kg dose, consistent with levels of ALX148 in the serum. These results indicate the CD47 antigen sink is saturable and that nonlinear increases in exposure and high coverage of CD47 in the tumor can be attained at higher doses of ALX148. These results also corroborated the effects described above of ALX148 on immune cells in the spleen and tumor compartment.

### ALX148 produces full target occupancy with an acceptable PK profile and has a favorable safety profile in non-human primates

As ALX148 binds cynomolgus monkey CD47 with high affinity, this species was used to assess the preclinical safety of ALX148. Potential toxicity of ALX148 was assessed in all tissues, including blood cells, in a 4 week repeat dose toxicity study. Cynomolgus monkeys were treated by weekly intravenous injection at doses of 0 (vehicle control), 10, 30, and 100 mg/kg for 4-consecutive weeks, followed by 4 weeks of recovery period in subset of animals at 0, 10, and 100 mg/kg. No toxicity or adverse findings related to CD47 blockade by ALX148 were observed in clinical observations, body weights, ophthalmic examinations, cytokine analysis, ECG parameters, clinical pathology including hematological analysis of red blood cells, white blood cells, and platelets, and anatomical pathology assessment. The no-observable-adverse-effect-level (NOAEL) in the study was the highest tested dose of 100 mg/kg. In a separate study, pharmacokinetics and target occupancy of ALX148 were assessed after a single dose of 10 or 30 mg/kg intravenous injection when given together with a single intravenous dose of 0.05 mg/kg rituximab. Systemic exposure to ALX148 increased in a nonlinear manner with a dose increase from 10 to 30 mg/kg (Figure A in S5 Fig) and complete target occupancy was observed in circulating T lymphocytes within 4 hours of dosing at both dose levels. The higher dose of 30 mg/kg conferred longer persistence of CD47 occupancy (12 days) compared to 10 mg/kg (7 days) (Figure B in S5 Fig).

## Discussion

The therapeutic blockade of immune checkpoints has brought dramatic advances in anticancer therapy, leading to enhanced antitumor immunity and sustained clinical responses[2–4]. The checkpoint inhibitors in clinical use act on adaptive immunity, specifically by enhancing antitumor T cell responses. The CD47-SIRPα axis is an additional checkpoint that acts upon innate immunity by suppression of myeloid cell function, and several molecules to block this interaction have entered clinical development [40]. Here, we describe the development of ALX148, an engineered high affinity SIRPα-Fc fusion protein that blocks the CD47-SIRPα interaction and induces antitumor immunity by bridging innate and adaptive immune responses while maintaining a favorable preclinical safety profile.

The majority of therapeutics in development to target CD47 bind both CD47 and, to varying extents, Fcγ receptors. Thus, these molecules induce phagocytosis both by blocking the interaction of CD47 with SIRPα and by providing a positive signal to macrophages through their Fcγ receptors. Because CD47 is also expressed at high levels on normal healthy cells, such as platelets and erythrocytes, these molecules have the potential to induce phagocytosis of non-tumor cells and result in toxicity. In fact, early clinical and preclinical studies have described thrombocytopenia and anemia subsequent to treatment with these molecules [31, 41, 42].

ALX148 was developed based upon the finding that phagocytosis can be enhanced by a combination approach in which CD47 blockade and Fcγ receptor engagement come from different molecules [32]. We hypothesized that CD47 blockade with a molecule containing an inactive Fc domain could enhance the activity of antitumor antibodies while maintaining a favorable safety profile. ALX148 specifically binds to CD47 with picomolar affinity and blocks the binding of wild-type SIRPα to CD47 on cells. However, the modified Fc domain in ALX148 is devoid of binding to Fcγ receptors and complement C1q. When ALX148 is used in combination with tumor-specific antibodies, the active Fc domains of the latter will engage macrophage Fc receptors, thereby providing a pro-phagocytosis signal in a targeted manner. Thus, the inadvertent targeting of normal CD47-expressing cells should be avoided and the potential for treatment-related toxicity is minimized.

Consistent with our hypothesis, the preclinical safety profile of ALX148 differentiates it from other CD47-blocking molecules in development. In contrast to the anti-CD47 antibodies 5F9 [30], B6H12, or CC2C6, ALX148 does not cause hemagglutination of human erythrocytes *in vitro*. ALX148 also has no *in vitro* ADCC or C1q binding activity. As a single agent, ALX148 did not stimulate phagocytosis by human macrophages, confirming the elimination of functional interactions with activating macrophage Fcγ receptors. Furthermore, in a mouse hematology study, no effects on RBC, WBC, or platelets were seen after ALX148 administration, while a control molecule possessing an active Fc domain caused an acute depletion of these cells. Most importantly, no toxicity or adverse findings related to CD47 blockade by ALX148 were observed up to the highest treatment dose of 100 mg/kg in a repeat-dose nonhuman primate toxicity study. This included the absence of any adverse observations related to platelets or erythrocytes.

Although ALX148 does not itself possess an active Fc domain, it enhances the activity of antitumor antibodies. This is similar to previously reported CD47 blocking agents that utilize the SIRPα D1 domain [32]. In *in vitro* phagocytosis assays, ALX148 enhanced the ADCP activity of anti-cancer antibodies that contain an active Fc, such as trastuzumab, cetuximab, daratumumab, and obinutuzumab, in a dose-dependent manner. Furthermore, ALX148 enhanced the antitumor activity of obinutuzumab, trastuzumab, and rituximab in murine xenograft models of tumorigenesis using the human Z138, OE19, and Raji cell lines, respectively. Because this xenograft model was established in NOD-SCID mice lacking most functional immune cell subsets except myeloid cells, these results demonstrate that ALX148 enhances the antitumor efficacy of myeloid cells *in vivo*. CD47 blockade can also enhance neutrophil-mediated ADCC of cells bound by an active Fc [6, 60]. Thus, ADCC may contribute to the efficacy observed for ALX148 and antitumor antibody combinations in xenograft models. Together, these results support the use of ALX148 in combination with a wide range of anti-cancer antibodies.

In addition, our studies demonstrated that blocking the CD47-SIRPα interaction enhances the adaptive immune response, which is in agreement with an increasing body of evidence. ALX148 binds to murine CD47 with a K_D_ of 14 nM, a high affinity that is more than 300-fold greater than the affinity of mouse SIRPα for mouse CD47 [61]. This potent binding allows investigation of both antitumor activity and mechanism in immunocompetent mice. Previous studies have relied upon *in vitro* differentiated macrophages and DCs to demonstrate that in response to CD47 blockade these cells process tumor antigen and present them for priming of cytotoxic T cells [34, 36]. In contrast, the present study directly interrogates the *in vivo* cellular response to blocking CD47.

We show that ALX148 alone or in combination with anti-PD-1 results in increased activation of both CD8^+^ and CD8^-^ DCs in the spleen. This is independent of Fcγ receptors, drawing a distinction between DC activation and the enhancement of macrophage phagocytosis. DC activation is detectable as early as four hours (data not shown) and as long as ten days after a single treatment with ALX148. In addition, there was a significant increase in CD8^+^ DCs in the spleen of mice treated with ALX148 in combination with anti-PD-1. CD8^+^ DCs are the primary DC subset responsible for cross-priming of cytotoxic T cells [62]. Consistent with this role, increased cytotoxic CD8^+^ T cell numbers and cytokine production were seen with ALX148 treatment. Interestingly, treatment of mice with ALX148 resulted in a decrease in splenic CD8^-^ DC numbers that continued ten days after treatment. The mechanism for this decrease remains unclear. As the up-regulation of activation markers such as CD86 was observed in this population, one possibility is that this DC subset is particularly sensitive to activation-induced cell death. Conventional DCs have a half-life of 3–6 days [63], and ALX148 occupancy of CD47 on splenic CD4+ T cells persists beyond eight days when dosed at 10 mg/kg. Therefore, it is possible that CD8^-^ DCs would replenish at time points beyond the ten days examined in our study. Although the role of CD8^-^ DCs is not well characterized, they are thought to be superior to CD8^+^ DCs for CD4^+^ T cell priming [63]. In our system, however, the decrease in CD8^-^ DCs did not correlate with effects on the CD4^+^ T cell population, since an increase in both effector and memory T cells was observed in the spleen. Collectively, our data show that in the spleen of mice, ALX148 induces DC activation as a single agent and that the combination of ALX148 and anti-PD-1 mediates increased numbers of CD8^+^ DCs, a cell population that primes cytotoxic T cells for antitumor activity. A topic of current interest is the mechanism by which inhibition of the CD47-SIRPα interaction mediates DC activation. Cytosolic DNA sensing by the STING pathway has been implicated in this process [34, 38], and investigations of the effect of ALX148 on this pathway are ongoing.

Within the tumor microenvironment, macrophages are phenotypically heterogeneous and have different functional properties with regard to tumorigenesis. While the functional classification of tissue-resident macrophages represents extremes of a continuum that is shaped by the tumor microenvironment, there is evidence to support broad definitions of function. M1 (classically activated) macrophages have been shown to exhibit strong tumoricidal activity, while M2 (alternatively activated) macrophages are thought to have immunosuppressive functions and to mediate tumor progression [64–67]. To extend our understanding of ALX148 function beyond confirming the enhancement of macrophage phagocytosis, effects on TAM populations were investigated in detail. Treatment with ALX148 and anti-PD-1 resulted in an increased M1/M2 ratio among TAMs. A similar skewing of TAMs toward an M1-like phenotype subsequent to antibody-mediated CD47-SIRPα disruption has been described previously [68]. TAM repolarization is apparently independent of which arm of the CD47-SIRPα interaction is targeted, as this phenotype was also described for mice treated with an antibody against SIRPα in a RENCA tumor model [69]. This less suppressive phenotype is consistent with enhanced tumoricidal activity. Furthermore, in the tumors of mice treated with ALX148 and anti-PD-1, we observed decreases in both mMDSC and Tregs that were not evident under monotherapy. This reduction in immunosuppressive cells establishes a milieu for effective tumor elimination by T cells. The combination leads to an increase in IFNγ expressing CD8+ T cells upon stimulation while the number of tumor-specific CD8+ T cells remained similar, as in a previously reported study of checkpoint inhibitor combinations [57].

In the CT26 colon carcinoma model, TIL phenotyping was conducted following a single dose of test articles with the goal of elucidating ALX148-driven cellular responses. These results demonstrate that in combination with anti-PD-1, ALX148 enhances antitumor efficacy and the persistence of antitumor immune responses. In the MC38 colon carcinoma model, multiple doses of ALX148 and anti-PD-L1 demonstrated even stronger antitumor efficacy and a more robust cellular immune response within the tumor than that produced by a single dose of ALX148 and anti-PD-1 in the CT26 model. Additional studies are needed to elucidate the basis for the heightened cellular response observed in the MC38 model. The second dose of ALX148 in combination with anti-PD-L1, the decreased interval between dosing and tumor harvest, or the variability in anti-tumor activity observed in different models may be responsible for the improved response. Alternatively, the murine IgG1 Fc of the anti-PD-L1 antibody used here could contribute to efficacy by opsonizing tumor cells for killing. Further exploration of this would be necessary, as there have been differing reports on the effector activity of the murine IgG1 Fc [70, 71]. Nevertheless, ALX148 in combination with either anti-PD-1 or anti-PD-L1 is capable of driving an enhanced anti-tumor cellular response.

The widespread expression of CD47 poses a challenge to the therapeutic targeting of this molecule because of the effect this large antigen sink will have on pharmacokinetics. Rapid clearance of the 17 kD SIRPα D1 domain would largely hinder its therapeutic use in humans, despite the ability to potently block the CD47-SIRPα interaction. With ALX148, fusion of this SIRPα domain to a modified human IgG1 Fc confers increased molecular mass and an interaction with the neonatal Fc receptor, both of which are expected to extend pharmacokinetics.

Studies of ALX148 pharmacokinetics in mice and cynomolgus monkeys demonstrated a nonlinear increase in ALX148 levels with increasing doses, consistent with the presence of a saturable CD47 antigen sink. In mice, higher doses of ALX148 led to increased tumor penetration, with more CD47 occupancy on tumor cells and tumor-infiltrating lymphocytes. Comparison of CD47 occupancy at 10 and 30 mg/kg demonstrated that greater levels of ALX148 are required to attain full occupancy in the tumor than are required for the periphery. Importantly, these effects were observed at well-tolerated doses of ALX148.

In both mice and monkeys, higher doses of ALX148 also led to a longer duration of CD47 occupancy. In fact, full CD47 occupancy on peripheral blood cells was maintained for twelve days after administration of cynomolgus monkeys with a single ALX148 dose at 30 mg/kg, with seven day occupancy observed after a 10 mg/kg dose. The pharmacokinetic and pharmacodynamic properties of ALX148 are consistent with that of an anti-CD47 antibody observed in NHP [31] and support a weekly or biweekly dosing frequency in human patients. Notably, ALX148 is smaller than a typical antibody, with about one-half the molecular weight. This small size may provide the additional advantages of better tissue penetration and twice the number of molecules at the same dosage level.

The CD47-SIRPα axis is now recognized as an important innate immune checkpoint in cancer. Here, we describe the therapeutic targeting of this pathway with ALX148, which was designed to potently block the CD47-SIRPα while avoiding toxicity to normal cells. ALX148 induces innate and adaptive immune responses, enhances preclinical anti-tumor activity in combination with a variety of cancer therapies, and has a favorable preclinical safety profile. It is currently being investigated in a first-in-patient, phase 1 dose escalation/expansion, multi-center study in patients with advanced solid tumors and lymphoma. (NCT03013218).

## Supporting information

S1 Fig ALX148 has no activity in assays for ADCC activity and C1q binding. (A) Cell-based ADCC activity assay. Target and effector cells were incubated with the indicated amounts (x-axis) of different proteins. Luminescence, resulting from FcγRIIIa receptor signaling, is indicated on the y-axis. Samples were run in duplicate and averages of the duplicate values were graphed with error bars displaying standard deviation. (B) ELISA assay to measure binding of recombinant human C1q to the indicated proteins. Quantity of C1q protein is indicated on the x-axis. Optical density resulting from C1q binding is indicated on the y-axis. Samples were run in duplicate and the average of the duplicate values is graphed with error bars showing standard deviation.
(TIF)

S2 Fig Fluorescence microscopy to detect phagocytosis. *In vitro* phagocytosis experiment with human monocyte-derived macrophages and CFSE-labeled DLD-1 cells treated with 100nM ALX148 and 100 ng/mL of cetuximab for two hours were washed with PBS and fixed on slides. Cells were imaged using immunofluorescence microscopy to detect phagocytosis. Bright field (A), CFSE-immunofluorescence (B), and merged images showing CFSE-labeled DLD-1 inside macrophages as indicated by arrows (C).
(TIF)

S3 Fig ALX148 enhances antitumor therapy *in vivo*. (A) Raji B-cell lymphoma cells were implanted subcutaneously on the right flanks of NOD-SCID mice. Mice with established tumors (average of 85 mm^3^) were randomized and treated intraperitoneally with vehicle, ALX148, rituximab, or ALX148 + rituximab. Left panel shows mean tumor growth ± SEM of n = 10 mice and right panel shows survival curves. ALX148 in combination with rituximab showed significant inhibition of tumor growth as compared to rituximab alone, ****p<0.0001 on day 26 and significant increased survival as compared to PBS alone (****p<0.0001, log-rank (Mantel-Cox) test). Mice treated with rituximab alone also had increased survival as compared to PBS, (**p<0.01, log-rank (Mantel-Cox) test). (B) CT26 colon carcinoma cells were implanted subcutaneously on the right flanks of BALB/C mice. When tumors reached an average of 77 mm^3^, mice were randomized into groups and treated i.p. with PBS, ALX148, anti-4-1BB or ALX148 + anti-4-1BB. Graph shows survival curves of n = 10 mice per group. ALX148 in combination with anti-4-1BB and anti-4-1BB groups showed significant increased survival as compared to PBS alone (**p<0.01 and *p<0.05, log-rank (Mantel-Cox) test). Results are representative of two independent experiments.
(TIF)

S4 Fig AH-1 specific CD8^+^ T- cell response in CT26 and not MC38 tumor-bearing mice. Spleen of naïve BALB/c, CT26 and MC38 tumor bearing mice, 15 days post implantation. Cell suspension were stained directly with AH-1 MHC I tetramer to identify antigen-specific CD8^+^ T cells and percent AH-1^+^CD8^+^ of CD3^+^ T cells are shown. (A) Spleens were harvested 10 days post single dose of PBS from naïve BALB/c, CT26 and MC38 tumor-bearing mice. CT26 tumor-bearing mice show significant increase in AH-1 specific CD8+ T cells compared to both naive BALB/c and MC38 tumor-bearing mice. (B) Spleens were harvested 10 days post single dose of PBS or ALX148 + anti-PD-1 from naive C57BL/6 and MC38 tumor-bearing mice. Mice treated with ALX148 in combination with anti-PD-1 do not show AH-1 specific CD8^+^ T cells. Results are representative of one experiment, n = 4–5 mice/group, **p<0.01. Statistics were performed using One-Way ANOVA, Tukey-Kramer.
(TIF)

S5 Fig Pharmacokinetic and pharmacodynamic parameters of ALX148 in cynomolgus monkeys. (A) Serum concentration of ALX148 and (B) occupancy of CD47 by ALX148 at the indicated time points in monkeys administered 30 mg/kg (black lines) or 10 mg/kg (grey lines) ALX148. Curves for individual monkeys are shown. Vertical dashed lines indicate infusion of monkeys on day 1.
(TIF)

S1 Table Apparent affinity of ALX148 for human FcRn receptor. The apparent affinity of ALX148 for human FcRn receptor was determined using SPR as described in Materials and Methods. ALX222, which has a wildtype human IgG1 Fc domain was used as a positive control. The binding affinity of ALX148 for human FcRn is comparable to that of ALX222.
(DOCX)
