# Supplementary figures and images for: ALX148 blocks CD47 and enhances innate and adaptive antitumor immunity with a favorable safety profile
Source: PLoS One. 2018 Aug 22;13(8):e0201832. doi: 10.1371/journal.pone.0201832 (PMC6104973; doi:10.1371/journal.pone.0201832)

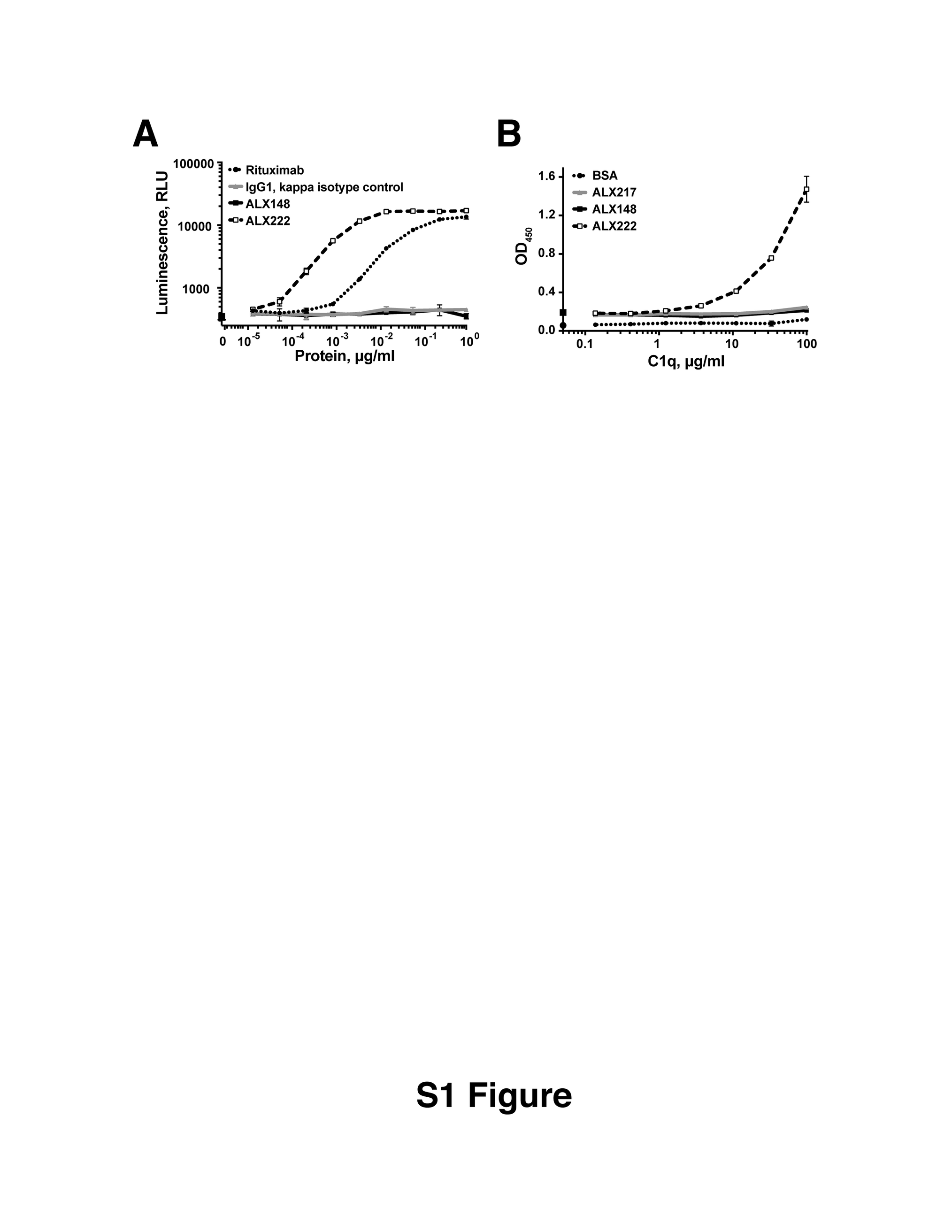

Supplement: S1 Fig — (A) Cell-based ADCC activity assay. Target and effector cells were incubated with the indicated amounts (x-axis) of different proteins. Luminescence, resulting from FcγRIIIa receptor signaling, is indicated on the y-axis. Samples were run in duplicate and averages of the duplicate values were graphed with error bars displaying standard deviation. (B) ELISA assay to measure binding of recombinant human C1q to the indicated proteins. Quantity of C1q protein is indicated on the x-axis. Optical density resulting from C1q binding is indicated on the y-axis. Samples were run in duplicate and the average of the duplicate values is graphed with error bars showing standard deviation. (TIF) [file pone.0201832.s001.tif]

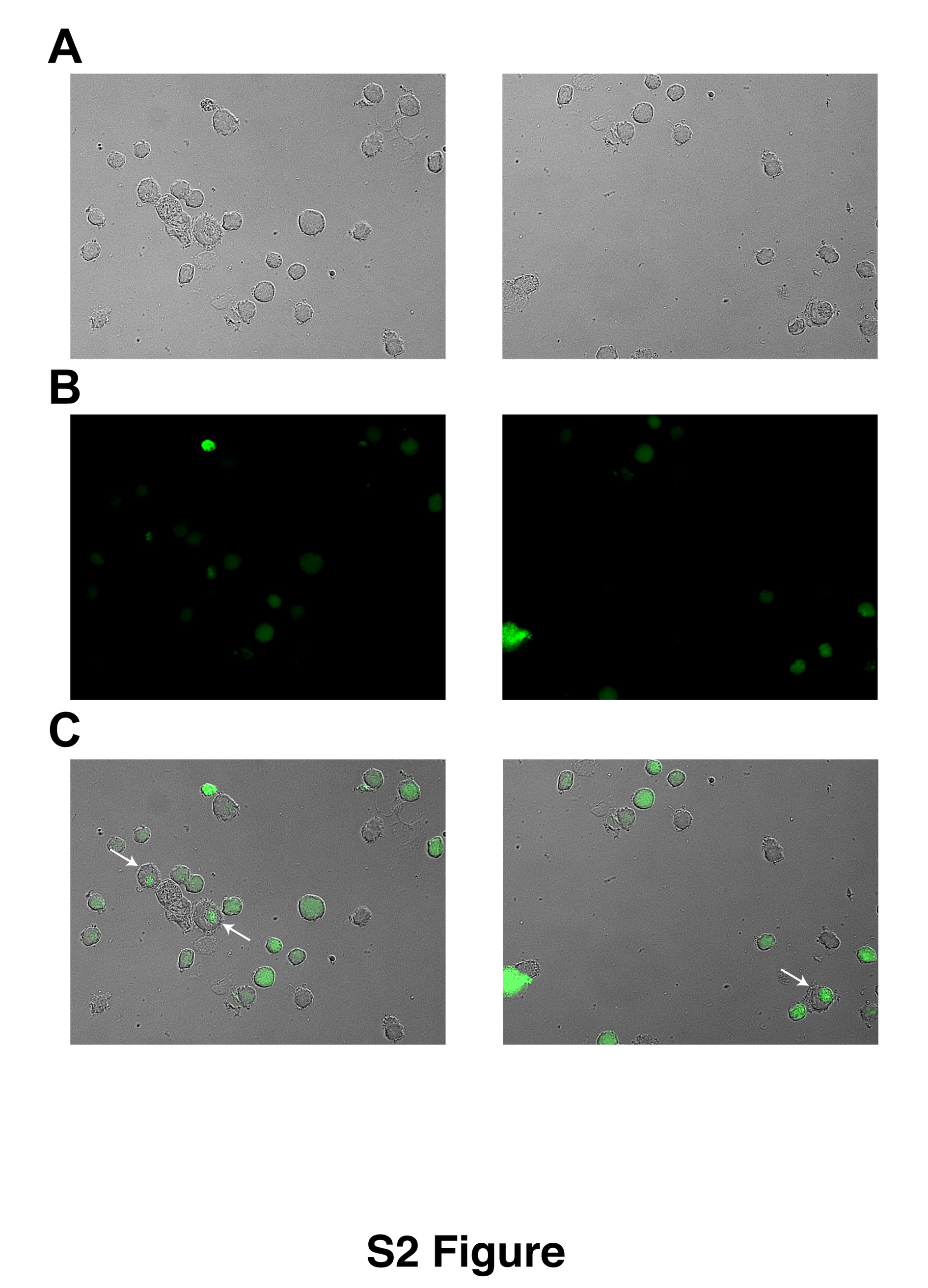

Supplement: S2 Fig — In vitro phagocytosis experiment with human monocyte-derived macrophages and CFSE-labeled DLD-1 cells treated with 100nM ALX148 and 100 ng/mL of cetuximab for two hours were washed with PBS and fixed on slides. Cells were imaged using immunofluorescence microscopy to detect phagocytosis. Bright field (A), CFSE-immunofluorescence (B), and merged images showing CFSE-labeled DLD-1 inside macrophages as indicated by arrows (C). (TIF) [file pone.0201832.s002.tif]

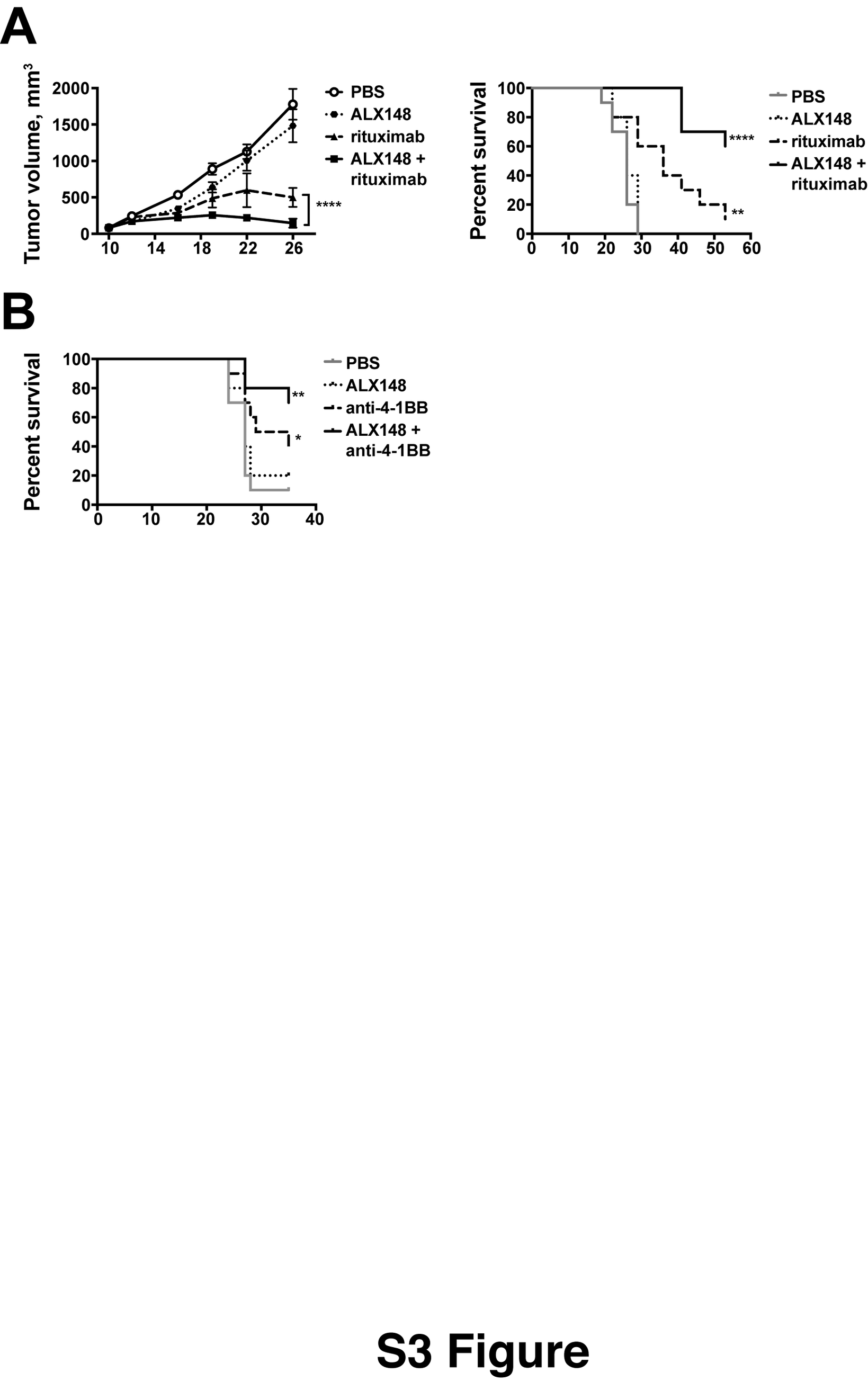

Supplement: S3 Fig — (A) Raji B-cell lymphoma cells were implanted subcutaneously on the right flanks of NOD-SCID mice. Mice with established tumors (average of 85 mm3) were randomized and treated intraperitoneally with vehicle, ALX148, rituximab, or ALX148 + rituximab. Left panel shows mean tumor growth ± SEM of n = 10 mice and right panel shows survival curves. ALX148 in combination with rituximab showed significant inhibition of tumor growth as compared to rituximab alone, ****p<0.0001 on day 26 and significant increased survival as compared to PBS alone (****p<0.0001, log-rank (Mantel-Cox) test). Mice treated with rituximab alone also had increased survival as compared to PBS, (**p<0.01, log-rank (Mantel-Cox) test). (B) CT26 colon carcinoma cells were implanted subcutaneously on the right flanks of BALB/C mice. When tumors reached an average of 77 mm3, mice were randomized into groups and treated i.p. with PBS, ALX148, anti-4-1BB or ALX148 + anti-4-1BB. Graph shows survival curves of n = 10 mice per group. ALX148 in combination with anti-4-1BB and anti-4-1BB groups showed significant increased survival as compared to PBS alone (**p<0.01 and *p<0.05, log-rank (Mantel-Cox) test). Results are representative of two independent experiments. (TIF) [file pone.0201832.s003.tif]

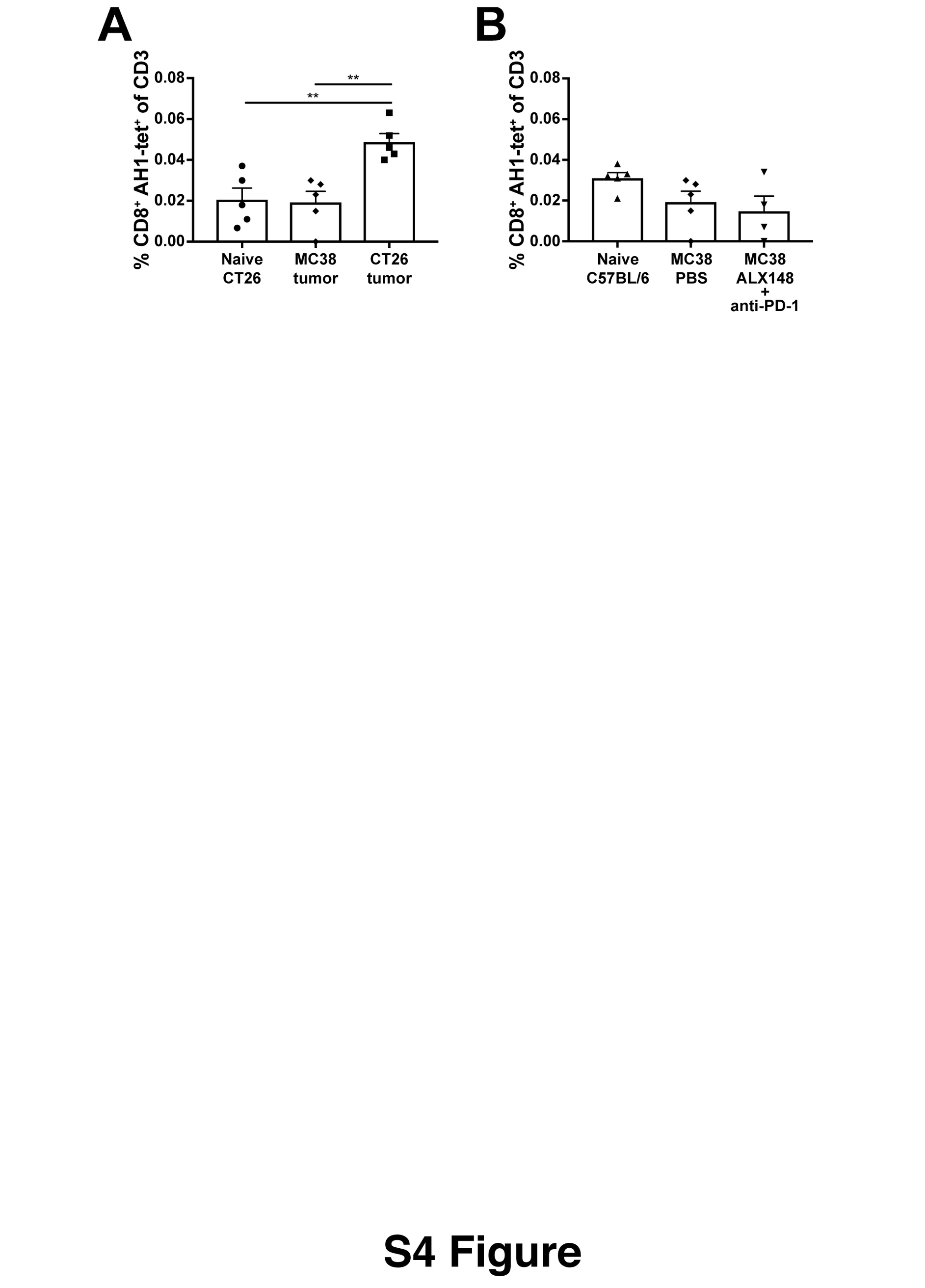

Supplement: S4 Fig — Spleen of naïve BALB/c, CT26 and MC38 tumor bearing mice, 15 days post implantation. Cell suspension were stained directly with AH-1 MHC I tetramer to identify antigen-specific CD8+ T cells and percent AH-1+CD8+ of CD3+ T cells are shown. (A) Spleens were harvested 10 days post single dose of PBS from naïve BALB/c, CT26 and MC38 tumor-bearing mice. CT26 tumor-bearing mice show significant increase in AH-1 specific CD8+ T cells compared to both naive BALB/c and MC38 tumor-bearing mice. (B) Spleens were harvested 10 days post single dose of PBS or ALX148 + anti-PD-1 from naive C57BL/6 and MC38 tumor-bearing mice. Mice treated with ALX148 in combination with anti-PD-1 do not show AH-1 specific CD8+ T cells. Results are representative of one experiment, n = 4–5 mice/group, **p<0.01. Statistics were performed using One-Way ANOVA, Tukey-Kramer. (TIF) [file pone.0201832.s004.tif]

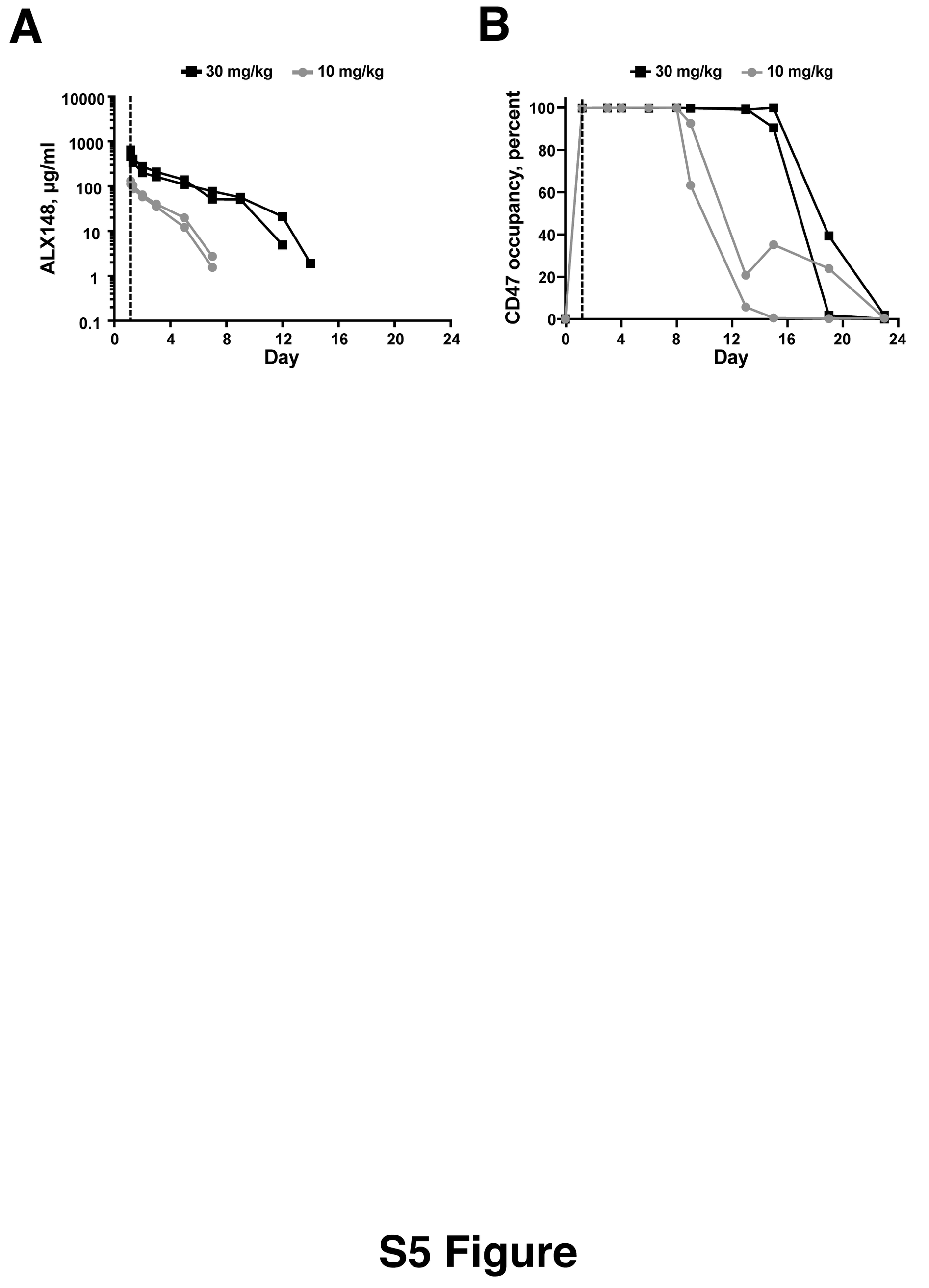

Supplement: S5 Fig — (A) Serum concentration of ALX148 and (B) occupancy of CD47 by ALX148 at the indicated time points in monkeys administered 30 mg/kg (black lines) or 10 mg/kg (grey lines) ALX148. Curves for individual monkeys are shown. Vertical dashed lines indicate infusion of monkeys on day 1. (TIF) [file pone.0201832.s005.tif]
