# Supplementary material for: ALX148 blocks CD47 and enhances innate and adaptive antitumor immunity with a favorable safety profile
Source: PLoS One. 2018 Aug 22;13(8):e0201832. doi: 10.1371/journal.pone.0201832 (PMC6104973; doi:10.1371/journal.pone.0201832)
Supplement: S1 Table — The apparent affinity of ALX148 for human FcRn receptor was determined using SPR as described in Materials and Methods. ALX222, which has a wildtype human IgG1 Fc domain was used as a positive control. The binding affinity of ALX148 for human FcRn is comparable to that of ALX222. (DOCX) [file pone.0201832.s006.docx]

**S1 Table: Apparent affinity of ALX148 for human FcRn receptor**

| **Protein** | **hFcRn KD (M), pH 5.8** | **SD** | **N** |
| --- | --- | --- | --- |
| ALX222 | 4.5E-07 | 9.5E-08 | 4 |
| ALX148 | 5.8E-07 | 1.0E-07 | 3 |

N, number of independent experiments; SD, standard deviation of results from independent experiments
